# Intersectionality in the mortality of Brazilians aged 50 years or older: analysis of ELSI-Brasil

**DOI:** 10.1590/0102-311XEN244825

**Published:** 2026-10-12

**Authors:** Luiza Oliveira-Santos, Maria Fernanda Lima-Costa, Taynãna César Simões

**Affiliations:** 1 Instituto René Rachou, Fundação Oswaldo Cruz, Fiocruz, Belo Horizonte, Brasil.; 2 Núcleo de Estudos em Saúde Pública e Envelhecimento, Fundação Oswaldo Cruz, Fiocruz/Universidade Federal de Minas Gerais, Belo Horizonte, Brasil.

**Keywords:** Aging, Mortality, Intersectionality, Envejecimiento, Mortalidad, Interseccionalidad

## Abstract

The objective was to analyze the factors associated with all-cause mortality and cause-specific mortality groups, including garbage codes (GC), from an intersectional perspective (operationalized by the combination of sex, race/skin color, education, and income), in a cohort of Brazilians aged 50 years or older. This is a prospective cohort study with data from 8,918 participants of the *Brazilian Longitudinal Study of Aging* (ELSI-Brasil), followed between 2015 and 2021. Intersectionality was operationalized in 32 strata. Mortality heterogeneity was quantified by the Multilevel Analysis of Individual Heterogeneity and Discriminatory Accuracy (MAIHDA). The association with time until death was analyzed using traditional and frailty Cox proportional hazards models, adjusted for sociodemographic, health, and behavioral variables. There were 817 deaths. The MAIHDA model without adjustment variables indicated that 7% of the mortality variation was attributable to differences between intersectional strata. In the final model adjusted with frailty, the adjustment variables explained part of the risk, but the residual analysis showed that strata composed of women with low education maintained a high residual mortality risk. In contrast, high-income non-white men presented a lower risk than expected. Intersectionality is a structural determinant of mortality. The intersection of axes of oppression generates risks that are not reducible to the sum of their parts, demanding policies that act on the social structures that produce inequity.

## Abstract (es)

Este estudio tuvo como objetivo analizar los factores asociados a la mortalidad general y la mortalidad por grupos de causas específicas, incluidos los códigos *garbage* (CG), desde la perspectiva de la interseccionalidad (operacionalizada mediante la combinación de sexo, raza/color de la piel, escolaridad e ingresos), en una cohorte de brasileños de 50 años o más. Estudio de cohorte prospectivo con datos de 8.918 participantes del *Estudio Brasileño Longitudinal del Envejecimiento* (ELSI-Brasil), seguidos entre 2015 y 2021. La interseccionalidad se operacionalizó en 32 estratos. La heterogeneidad en la mortalidad se cuantificó mediante el Análisis Multinivel de Heterogeneidad Individual y Precisión Discriminatoria (MAIHDA). La asociación con el tiempo hasta la muerte se analizó a través de modelos de riesgos proporcionales de Cox tradicional y con fragilidad, ajustados por variables sociodemográficas, de salud y de comportamiento. Se registraron 817 muertes. El modelo MAIHDA sin variables de ajuste indicó que el 7% de la variación en la mortalidad era atribuible a las diferencias entre los estratos interseccionales. En el modelo final ajustado con fragilidad, las variables de ajuste explicaron parte del riesgo, sin embargo, el análisis de los residuos reveló que los estratos compuestos por mujeres con baja escolaridad mantuvieron un alto riesgo de mortalidad residual. En cambio, los hombres no blancos de altos ingresos presentaron un riesgo inferior al esperado. La interseccionalidad es un determinante estructural de la mortalidad. La superposición de ejes de opresión genera riesgos que no se pueden reducir a la suma de sus partes, lo que exige políticas que actúen sobre las estructuras sociales que producen inequidad.

## Introduction

Population aging is a global demographic phenomenon that, in Brazil, is occurring rapidly and in a context of deep and persistent social inequalities, posing unique challenges to public health ^1^. Current scientific knowledge indicates that the country is experiencing a complex and non-linear epidemiological transition, characterized by a triple burden of diseases: the growing prevalence of chronic non-communicable diseases (NCDs); the persistence of an unresolved agenda of infectious and nutritional diseases; and the maintenance of high rates of mortality from external causes ^2^ ^,^ ^3^.

It is known that older adult mortality is not homogeneously distributed, being influenced by a complex interrelation of socioeconomic, demographic, and health factors accumulated throughout life ^4^. However, a persistent gap in the literature is the tendency to analyze these factors in isolation, which is insufficient to capture the complexity of health inequities. This study is motivated by the following research question: how does the intersection of multiple axes of social oppression (gender, race/skin color, social class) influence all-cause and cause-specific mortality risk in older adults in Brazil?

To answer this question, this study is based on the theoretical model of intersectionality. Named by Kimberlé Crenshaw ^5^ in 1989, this framework, discussed by Brazilian intellectuals such as Lélia Gonzalez ^6^ and Sueli Carneiro ^7^, argues that oppressions do not act additively, but rather reinforce one another, producing inequities that are greater than the sum of their parts.

International studies show that individuals from more recent cohorts, such as baby boomers, present a higher multimorbidity burden than previous generations, with inequalities accentuated by race and gender ^8^. In Brazil, Black older adults, especially women, are more likely to have worse intrinsic capacity - physical and mental decline - when compared to white men ^9^, demonstrating how intersectionality influences aging. Another gap that justifies this investigation is the methodological gap: the high prevalence of poorly defined causes of death or garbage codes (GC) in Brazilian official records compromises the reliability of analyses and policy planning, underestimating the disease burden in vulnerable groups ^10^ ^,^ ^11^.

Considering the above, the objective of this study was to analyze the factors associated with all-cause mortality and cause-specific mortality groups, including GCs, considering the role of intersectionality in risk inequalities and these associations, based on a nationally representative cohort of Brazilians aged 50 years and older, between 2015 and 2021.

## Materials and methods

### Study design and population

This is a prospective cohort study that used data from the *Brazilian Longitudinal Study of Aging* (ELSI-Brasil), a comprehensive investigation into the aging process in the country. ELSI-Brasil employs a complex sampling design, with stratification of municipalities, and selection of census sectors and households to ensure national representativeness. Full details on the ELSI-Brasil methodology were previously published ^12^.

Participants were recruited at baseline of the study, between 2015 and 2016. The follow-up for the outcome of death was carried out until the second wave of data collection (2019-2021), with the observation period ended on December 31, 2021. Mortality data, including date and underlying cause of death, were obtained through probabilistic linkage of the ELSI-Brasil database with the Brazilian Mortality Information System (SIM, acronym in Portuguese) of the Brazilian Ministry of Health. The linkage procedure used personal identification variables (name, date of birth, and mother’s name) and included a manual review step for handling homonyms and confirming records, following the institutional protocols previously described ^4^ ^,^ ^12^. This procedure ensured the reliability of the data and the confidentiality of the participants.

The eligibility criteria for this analysis were: having participated in the ELSI-Brasil baseline and possessing complete information for the variables used in definition the intersectionality construct. The initial sample of ELSI-Brasil was 9,412 participants. After the exclusion of 494 individuals due to missing data in the exposure variables, the final analytical sample was composed of 8,918 participants.

### Outcome variables

The primary outcome was time until all-cause death (overall mortality). Follow-up time was calculated in days, from the date of the baseline interview to the date of death or the final follow-up date for censored participants. Additionally, deaths were classified into four mutually exclusive groups for specific cause analyses, based on the 10^th^ revision of the International Classification of Diseases (ICD-10). The first group, “Infectious-parasitic diseases, malnutrition, and reproductive problems”, included chapters I, X, XI, XII, and XIV. The second, “NCDs, neoplasms, and their risk factors”, covered chapters II, III, IV, VI, IX, and XIII. The third group, “External causes and mental/behavioral disorders”, was composed of chapters V and XX. Finally, the fourth, “Garbage Codes (GC)”, including chapter XVIII (Poorly defined causes) and other codes considered of little use for public health, according to the list from the study *Global Burden of Disease* (GBD) ^13^.

### Intersectional analytical strategy and adjustment variables

The intersectional perspective was adopted as a central analytical strategy, being operationalized by the combination of four social markers to form 32 intersectional strata: biological sex (female, male), self-reported race/skin color (white, non-white), education (categorized into four levels: no formal education, 1 to 4 years, 5 to 10, and 11 years or more), and household income per capita (grouped into “up to BRL 1,190.00” and “greater than BRL 1,190.00”).

The dichotomy of race/skin color into “White” and “non-White” (Brown, Mixed-race, Indigenous, and Yellow) is justified based on the concept of structural racism, which identifies whiteness as the normative reference of privilege ^7^ ^,^ ^14^. Although Indigenous persons (n = 42) and Yellow persons (n = 5) have distinct origins, the small sample size would prevent reliable isolated estimates, so they were grouped in the racial subordination pole.

The adjustment variables were selected based on the literature as potential confounders. These included sociodemographic and contextual characteristics, such as birth cohort (grouped into four periods from 1910 to 1966), age (continuous), geographic macrorregion (North, Northeast, South, Central-West, Southeast), place of residence (urban, rural), marital status (unmarried, married), and living alone (yes, no). Factors related to access to and use of healthcare services, such as having health insurance (yes, no) and household coverage by the Family Health Strategy - FHS (yes, no), were also considered.

Finally, health and behavioral condition variables were included: the number of self-reported morbidities (constructed based on the count of diagnoses of hypertension, back problem, high cholesterol, arthritis, depression, diabetes, heart problem, chronic obstructive pulmonary disease - COPD -, stroke, cancer, asthma, kidney failure, Parkinson’s, and Alzheimer’s), the occurrence of falls in the last year (yes, no), the presence of functional limitation in at least one of the basic activities of daily living (BADL) - dressing, eating, bathing, using the toilet, moving around (walking within a room), and getting out of bed -, alcohol use (yes, no), smoking habit (ex-smoker, current smoker, never smoked), and the level of physical activity practice (sufficient - if at least 150 minutes of physical activity per week was performed -; insufficient - if less than 150 minutes of physical activity per week was performed).

### Statistical analysis

Initially, we performed a descriptive analysis of sample characteristics and mortality proportions. The associations between the variables and mortality were tested using the chi-square test with Rao-Scott correction ^15^ and Mood’s median test for continuous age ^16^, incorporating sample weights and design effects. In addition, the Bayesian information criterion (BIC) was calculated to rank the significance of the variables and to determine the order in which they were included in the multiple models ^17^. To quantify heterogeneity in mortality attributable to social position, we adjusted a null model of Multilevel Analysis of Individual Heterogeneity and Discriminatory Accuracy (MAIHDA), i.e., without adjustment variables. This is a multilevel logistic regression model in which individuals (level 1) are nested within their respective intersectional strata (level 2). The intraclass correlation coefficient (ICC) was calculated based on this model to estimate the proportion of total variation in mortality attributable to differences between strata ^18^.

To analyze time-to-event data, Cox proportional hazards models were used, with measures of association estimated via hazard ratios (HR) and their respective 95% confidence intervals (95%CI). The central strategy of the analysis was the use of Cox frailty models were used, with measures of association estimated via frailty hazard ratios (FHR). In this statistical technique, “frailty” refers to a random effect assigned to the construct of intersectionality, formed by the strata of social position (combination of sex, race/skin color, education, and income), in order to capture the unobserved heterogeneity that influences mortality risk ^18^. This approach extends the traditional Cox model and provides a more precise estimation of the effects of covariates and the contextual effect of intersectionality ^19^.

The modeling strategy followed three steps: (1) univariate Cox analysis for all adjustment variables; (2) selection of variables with p < 0.20 for inclusion in the multiple model; (3) adjustment of the Cox model with final multiple frailty, considering a significance level of 5% (p < 0.05). The assumption of proportional hazards was verified graphically and through the Schoenfeld residuals analysis. The variable “physical activity practice” violated this assumption and was therefore used to stratify the final overall mortality model. The same analytical strategy was applied to each of the four groups of causes of death. However, for analyses by groups of specific causes, standard Cox models were used. The analyses were performed in R software (http://www.r-project.org), version 4.5.2. ELSI-Brasil was approved by the Research Ethics Committee of the Oswaldo Cruz Foundation, Minas Gerais (CAAE 34649814.3.0000.5091).

## Results

The final sample of 8,918 participants had a median follow-up period of 4.8 years (interquartile range: 4.1-5.4 years). During follow-up, there were 817 deaths. The distribution of causes of death showed that chronic NCDs and neoplasms (group 2) were responsible for 41.8% of deaths, followed by GC (33%), infectious and parasitic diseases (group 1) with 21.2%, and external causes (group 3) with 4.1%. The application of the MAIHDA model without adjustment variables, which assesses mortality variability solely among intersectional strata, showed that 7% (ICC = 0.07) of the total variation in the risk of death was attributable to differences among the 32 groups. This result indicates moderate but significant heterogeneity, suggesting that social position is a relevant predictor of mortality.

The univariate analysis (Table 1) showed a social gradient, in which factors such as male sex, low education, advanced age, not being married, having functional limitation, a higher number of morbidities, smoking, and insufficient physical activity were associated with a higher risk of death. However, the comparison between the standard Cox model (HR) and the Cox model with frailty (FHR), which considers intersectional heterogeneity, showed important nuances. Contextual variables, such as living in a rural area or in the North macrorregion, did not show a significant association in the standard model, but presented a statistically significant association as protective factors in the frailty model (FHR = 0.82; 95% confidence interval - 95%CI: 0.72-0.93 for rural area), although with overlap of the confidence intervals between the estimates (HR and FHR). Conversely, coverage by the FHS, observed as a protective factor in the standard model, lost its statistical significance after adjustment for intersectional frailty.

**Table 1:** Incidences and hazard ratios (HR) * from Cox proportional hazards and frailty models (FHR) * estimated for all-cause mortality, according to exposure and adjustment variables. *Brazilian Longitudinal Study of Aging* (ELSI-Brasil, 2015-2021).

Variables	Incidence in the sample (95%CI)	Incidence of deaths (95%CI)	p-value **	HR (80%CI)	FHR (80%CI)	p-value (PT ***)
Biological sex			0,012			-
Male	40.5 (38.3-43.0)	11.5 (10.2-12.9)		1.35 (1.23-1.47)	-	
Female (reference)	59.5 (57.3-62.0)	9.7 (8.3-11.0)			-	
Race/Skin color			0.542			-
Mixed-race	50.1 (46.7-54.0)	10.7 (9.2-12.2)		1.02 (0.93-1.12)	-	
Black	9.9 (8.6-11.0)	9.3 (6.9-11.7)		0.95 (0.80-1.11)	-	
Indigenous and Yellow	4.8 (1.5-15.0)	11.7 (8.8-14.7)		1.05 (0.81-1.35)	-	
Withe (reference)	35.2 (30.6-40.0)	10.2 (8.8-11.6)		-	-	
Race/Skin color dichotomized			0.728			
Non-White	35.2 (30.6-40.0)	10.5 (9.3-11.8)		1.01 (0.92-1.11)	-	
White (reference)	64.8 (59.8-69.0)	10.2 (8.8-11.6)		-	-	
Education (years)			< 0.001			-
11 or more	6.8 (5.8-8.0)	5.9 (3.5-8.3)		0.38 (0.30-0.47)	-	
5 to 10	34.8 (32.3-37.0)	6.3 (5.3-7.3)		0.38 (0.33-0.43)	-	
1 to 4	39.2 (37.4-41.0)	12.1 (10.7-13.6)		0.73 (0.66-0.82)	-	
No formal education (reference)	19.3 (16.7-22.0)	15.9 (13.1-18.6)			-	
*Per capita* household income (BRL) (quartiles)			0.018			
Greater than 1,190.00	24.9 (21.9-28.2)	8.7 (7.3-10.3)		0.80 (0.70-0.91)	-	
788.10 to 1,190.00	20.5 (18.3-23.0)	9.6 (7.8-11.3)		0.93 (0.81-1.07)	-	
425.10 to 788.00	29.5 (27.8-31.4)	12.0 (10.1-13.9)		1.10 (0.98-1.24)	-	
0.00 to 425.00 (reference)	25.0 (20.7-29.9)	11.0 (9.4-12.6)			-	
Grouped *per capita* income			0.018			
Greater than 1,190.00	24.9 (21.9-28.0)	8.7 (7.3-10.2)		0.78 (0.70-0.87)	-	
Up to 1,190.00 (reference)	75.1 (71.8-78.0)	11.0 (9.7-12.2)			-	
Age group (years)			< 0.001			0.150
< 60 (reference)	20.3 (18.2-22.0)	5.8 (4.6-7.1)		-	-	
60 to 69	32.6 (31.1-34.0)	7.2 (6.1-8.4)		1.11 (0.96-1.29)	1.09 (0.94-1.27)	
70 to 79	27.7 (25.9-30.0)	10.1 (8.5-11.9)		1.64 (1.41-1.90)	1.54 (1.32-1.80)	
80 to 89	15.6 (14.0-17.0)	18.7 (15.8-21.4)		3.28 (2.82-3.81)	2.92 (2.50-3.42)	
90 or more	3.9 (3.2-5.0)	30.5 (24.0-36.9)		5.25 (4.35-6.35)	4.51 (3.71-5.49)	
Age			< 0.001			0.131
Continuous	63 (56-72)	74 (64-80)		1.07 (1.07-1.08)	1.07 (1.07-1.08)	
Birth cohort			< 0.001			0.482
1910-1939	15.9 (14.1-18.0)	27.9 (24.6-31.2)		8.11 (6.98-9.42)	7.68 (6.57-8.97)	
1940-1949	25.9 (24.3-28.0)	11.3 (9.6-12.9)		2.87 (2.45-3.37)	2.80 (2.38-3.29)	
1950-1959	32.0 (30.5-34.0)	6.6 (5.5-7.7)		1.75 (1.49-2.05)	1.74 (1.48-2.04)	
1960-1966 (reference)	26.1 (23.8-29.0)	3.6 (2.7-4.5)				
Macrorregion of residence			0.065			0.041
North	13.6 (4.2-36.0)	10.2 (5.0-15.5)		0.89 (0.74-1.07)	0.82 (0.62-0.99)	
Northeast	28.0 (19.1-39.0)	12.9 (10.5-15.3)		1.21 (1.09-1.35)	1.07 (0.96-1.20)	
South	10.2 (5.5-18.0)	9.7 (7.9-11.4)		1.06 (0.92-1.22)	1.05 (0.91-1.21)	
Cnetral-West	14.3 (8.5-23.0)	10.2 (7.0-13.4)		0.99 (0.84-1.17)	0.94 (0.80-1.11)	
Southeast (reference)	33.9 (25.4-44.0)	8.8 (7.8-9.8)				
Residential zone			0.884			0.390
Rural	16.4 (11.0-24.0)	10.7 (8.9-12.4)		1.02 (0.90-1.15)	0.82 (0.72-0.93)	
Urban (reference)	83.6 (76.1-89.0)	10.4 (9.2-11.5)		-	-	
Marital status			< 0.001			0.028
Unmarried	48.2 (45.9-51.0)	13.7 (11.7-15.6)		1.81 (1.65-1.98)	2.00 (1.82-2.20)	
Married (reference)	51.8 (49.4-54.0)	7.4 (6.6-8.2)		-	-	
Living alone			0.431			0.580
Yes	14.7 (13.5-16.0)	11.3 (9.0-13.5)		1.28 (1.13-1.45)	1.30 (1.14-1.47)	
No (reference)	85.3 (84.0-86.0)	10.3 (9.1-11.4)		-	-	
Has health insurance			0.056			0.940
Yes	21.8 (18.5-25.0)	9.2 (7.6-10.8)		0.91 (0.82-1.01)	1.14 (1.01-1.28)	
No (reference)	78.2 (74.6-82.0)	10.7 (9.6-11.8)		-	-	
Home covered by FHS			0.093			0.270
No	26.6 (22.0-32.0)	9.4 (8.0-10.8)		0.85 (0.76-0.95)	0.95 (0.85-1.06)	
Yes (reference)	73.4 (68.3-78.0)	10.9 (9.5-12.2)		-	-	
Number of morbidities ^#^			0.012			0.320
None	9.7 (9.0-11.0)	4.6 (2.4-6.8)		0.56 (0.48-0.65)	0.52 (0.45-0.61)	
One	16.4 (15.3-18.0)	6.9 (4.8-9.0)		0.73 (0.65-0.82)	0.70 (0.63-0.79)	
Two or more (reference)	73.9 (72.4-75.0)	11.3 (9.7-12.8)				
Occurrence of fall in the last 12 months			0.150			0.460
Yes	24.5 (23.3-26.0)	11.6 (9.5-13.8)		1.30 (1.18-1.44)	1.32 (1.20-1.46)	
No (reference)	75.5 (74.4-77.0)	10.0 (9.0-11.1)		-	-	
Limitation in BADL ^##^			< 0.001			0.004
Yes	17.6 (16.3-19.0)	22.7 (19.0-26.4)		3.13 (2.85-3.44)	2.88 (2.62-3.17)	
No (reference)	82.4 (80.9-84.0)	7.8 (7.1-8.5)		-	-	
Smoking habit			< 0.001			0.160
Ex-smoker	39.1 (36.6-42.0)	12.0 (10.4-13.6)		1.51 (1.37-1.67)	1.35 (1.22-1.50)	
Curremt smoker	16.4 (15.4-17.0)	11.6 (9.4-13.7)		1.54 (1.36-1.75)	1.39 (1.22-1.58)	
Never smoked (reference)	44.6 (42.0-47.0)	8.6 (7.3-9.9)				
Alcohol use			< 0.001			0.061
Yes	23.9 (22.0-26.0)	7.3 (6.1-8.6)		0.68 (0.61-0.76)	0.71 (0.63-0.79)	
No (reference)	76.1 (74.0-78.0)	11.4 (10.1-12.7)		-	-	
Physical activity pratcice ^###^			< 0.001			< 0.001
Insufficient	34.3 (32.0-37.0)	18.1 (15.1-21.1)		2.49 (2.27-2.73)	2.33 (2.12-2.56)	
Sufficient (reference)	65.7 (63.3-68.0)	6.8 (6.0-7.6)		-	-	

80%CI: 80% confidence interval; 95%CI: 95% confidence interval; BADL: basic ativities of daily living; FHS: Family Health Strategy.

* HR refers to the univariate (crude) analysis considering the complex sample design; FHR refers to the univariate model with random effect (frailty) for the 32 intersectional strata, constructed based on the combination of sex, race/skin color, education, and income;

** p-value referring to weighted Kaplan-Meier test;

*** p-value of the prportional hazards test (presented only for the FHR fragility model);

^#^ Number of morbidities among self-reported conditions: systemic arterial hypertension; spinal problem; high cholesterol; arthritis or rheumatism; depression; diabetes; heart problem (angina, heart attack, heart failure); emphysema, chronic bronchitis, or chronic obstructive pulmonary disease (COPD); stroke; cancer; asthma; chronic kidney failure; Parkinson’s; and Alzheimer’s;

## Functional limitation: any difficulty in BADL - dressing, eating, bathing, using the toilet, moving around (walking within a room), and getting out of bed;

### Physical activity practice: sufficient - performing at least 150 minutes of physical activity per week; insufficient - performing less than 150 minutes of physical activity per week.

The analysis of residual effects for the multiple frailty model provided the quantification of residual variability in overall mortality risk among strata after controlling for adjustment factors (Table 2). The variance of the random effect (σ² = 0.08; p < 0.001) showed the persistence of groups with mortality risk above and below the sample average during the follow-up period, regardless of health conditions and individual behaviors. The strata that presented a significantly lower than expected mortality risk were: non-white men, high income, 5 to 10 years of education; and non-White men, high income, no formal education. In contrast, strata with risk significantly above the expected average included women with 1 to 4 years of education: non-White, low income; white, high income; and non-White, high income. Advanced age (FHR = 1.06 per year; 95%CI: 1.05-1.07), having functional limitation in BADLs (FHR = 1.99; 95%CI: 1.69-2.34), unmarried individuals (FHR = 1.48; 95%CI: 1.26-1.73), and being an ex-smoker (FHR = 1.36; 95%CI: 1.15-1.61) or current smoker (FHR = 1.93; 95%CI: 1.57-2.39) remained as independent risk factors. The absence of morbidities was a protective factor (FHR = 0.71; 95%CI: 0.55-0.91).

**Table 2:** Estimates of risk of all-cause mortality according to selected variables: Cox model with frailty (FHR) * adjusted for the construct of intersectionality. *Brazilian Longitudinal Study of Aging* (ELSI-Brasil, 2015-2021).

Variables	FHR	95%CI	p-value **
Marital status			
Married	Reference	Reference	Reference
Unmarried	1.48	1.26-1.73	< 0.001
Number of morbidities ***			
None	0.71	0.55-0.91	0.007
One	0.91	0.75-1.10	0.3
Two or more	Reference	Reference	Reference
Age			
Continuous	1.06	1.05-1.07	< 0.001
Limitation in BADL ^#^	Reference		
No	Reference	Reference	Reference
Yes	1.99	1.69-2.34	< 0.001
Smoking habit			
Never smoked	Reference	Reference	Reference
Ex-smoker	1.36	1.15-1.61	< 0.001
Smoker	1.93	1.57-2.39	< 0.001
Variance (σ^2^)	0.08	-	< 0.001

BADL: basic ativities of daily living; 95%CI: 95% confidence interval.

* Multiple Cox proportional hazards model with frailty for the 32 intersectional strata, constructed based on the combination of sex, race/skin color, education, and income;

** p-value referring to the Cox proportional hazards test;

*** Number of self-reported morbidities: systemic arterial hypertension; spinal problem; high cholesterol; arthritis or rheumatism; depression; diabetes; heart problem (angina, heart attack, heart failure); emphysema, chronic bronchitis, or chronic obstructive pulmonary disease (COPD); stroke; cancer; asthma; chronic kidney failure; Parkinson’s; and Alzheimer’s;

# Functional limitation: any difficulty in BADL - dressing, eating, bathing, using the toilet, moving around (walking within a room), and getting out of bed.

The univariate analysis by groups of causes of death showed distinct patterns. For group 1 (infectious diseases), older birth cohorts (HR = 9.27 for 1910-1939), living in the Northeast (HR = 1.39) or Central-West (HR = 1.56), not being married (HR = 2.02), and having functional limitation (HR = 2.95) were strong risk predictors (Table 3). For group 2 (chronic NCDs), in addition to social gradient by education, functional limitation (HR = 2.74) and smoking (HR = 1.57 for smokers) were prominent. In group 3 (external causes), the male sex, residing in rural areas (HR = 2.47), and especially smoking (HR = 4.14 for smokers) were prominent as the main risk factors. Finally, for deaths from GCs, functional limitation presented the highest risk among all those analyzed (HR = 4.07), along with advanced age and low education, indicating a profile of clinical and social vulnerability associated with the inaccuracy of the diagnosis of the cause of death.

**Table 3:** Mortality incidence and hazard ratios (HR) * from Cox proportional hazards and frailty models (FHR) * by grouping of the 10th revision of the International Classification of Diseases (ICD-10) and classification as garbage code (GC), according to exposure and adjustment variables. *Brazilian Longitudinal Study of Aging* (ELSI-Brasil, 2015-2021).

Variables	Group 1 **	Group 2 ***	Group 3 ^#^	GC
95%CI	95%CI	95%CI	95%CI
Null mode	2.2 (1.7-2.9)	4.4 (3.7-5.1)	0.4 (0.3-0.6)	3.4 (3.0-4.0)
Biological sex				
Male (reference)	2.4 (1.7-3.2)	4.8 (3.8-5.7)	0.7 (0.3-1.0)	3.7 (3.0-4.3)
Female	2.0 (1.3-2.8)	4.1 (3.2-4.9)	0.3 (0.1-0.4)	3.3 (2.7-3.9)
p-value ^##^	0.408	0.250	0.031	0.304
HR (80%CI)	0.78 (0.63-0.96)	0.70 (0.61-0.80)	0.39 (0.24-0.63)	0.84 (0.72-0.98)
Race/Skin color				
White (reference)	2.2 (1.5-3.0)	4.2 (3.4-5.1)	0.6 (0.3-0.8)	3.2 (2.4-3.9)
Non-White	2.2 (1.5-2.8)	4.4 (3.5-5.3)	0.4 (0.2-0.6)	3.6 (3.0-4.1)
p-value ^##^	0.856	0.706	0.252	0.384
HR (80%CI)	0.96 (0.77-1.19)	0.99 (0.86-1.13)	0.75 (0.47-1.17)	1.11 (0.95-1.30)
*Per capita* income (BRL)				
Up to 1,190.00 (reference)	2.3 (1.7-3.0)	4.5 (3.7-5.4)	0.5 (0.3-0.7)	3.7 (3.1-4.2)
Greater than 1,190.00	1.8 (1.0-2.6)	3.9 (2.8-5.0)	0.3 (0.0-0.6)	2.8 (2.0-3.5)
p-value ^##^	0.264	0.372	0.399	0.055
HR (80%CI)	0.74 (0.58-0.96)	0.89 (0.76-1.04)	0.38 (0.19-0.76)	0.73 (0.61-0.88)
Education (years)				
No formal eductaion (reference)	3.5 (2.0-4.9)	6.6 (4.5-8.7)	6.6 (4.5-8.7)	5.5 (4.2-6.8)
1 to 4	2.6 (1.5-3.6)	5.1 (4.1-6.2)	5.1 (4.1-6.2)	4.0 (3.2-4.8)
5 to 10	1.4 (0.8-1.9)	2.3 (2.1-3.4)	2.8 (2.1-3.4)	1.8 (1.2-2.3)
11 or more	0.9 (0.2-1.7)	1.7 (0.6-2.8)	1.7 (0.6-2.8)	3.2 (1.1-5.2)
p-value ^##^	0.007	< 0.001	0.724	< 0.001
HR (80%CI)				
1 to 4	0.68 (0.53-0.88)	0.80 (0.67-0.95)	1.29 (0.67-2.49)	0.66 (0.55-0.79)
5 to 10	0.37 (0.27-0.50)	0.43 (0.35-0.52)	0.81 (0.40-1.63)	0.30 (0.24-0.38)
11 or more	0.34 (0.20-0.58)	0.36 (0.26-0.51)	0.38 (0.09-1.56)	0.41 (0.29-0.58)
Age				
Continuous	73 (64-80)	71 (63-78)	62 (57-80)	77 (67-84)
p-value ^###^	< 0.001	< 0.001	0.900	< 0.001
HR (80%CI)	1.08 (1.07-1.09)	1.06 (1.06-1.07)	1.03 (1.00-1.05)	1.09 (1.08-1.10)
FHR (80%CI)	1.08 (1.07-1.09)	1.06 (1.06-1.07)	1.03 (1.01-1.05)	1.09 (1.08-1.10)
BIC	2,572.64	6,281.70	588.20	4,826.81
Birth cohort				
1910-1939	0.7 (0.3-1.0)	1.5 (0.8-2.1)	0.4 (0.0-0.7)	1.01 (0.7-1.5)
1940-1949	6.2 (4.5-7.8)	8.9 (6.4-11.5)	1.0 (0.4-1.6)	11.8 (9.5-14.1)
1950-1959	2.3 (1.3-3.2)	5.8 (4.6-7.1)	0.1 (0.0-0.3)	3.1 (2.3-3.9)
1960-1966 (reference)	1.4 (0.9-2.0)	3.2 (2.5-3.9)	0.5 (0.2-0.7)	1.5 (1.0-2.0)
p-value ^##^	< 0.001	< 0.001	0.017	< 0.001
HR (80%CI)				
1910-1939	9.27 (6.46-13.3)	7.15 (5.64-9.07)	3.05 (1.60-5.82)	9.76 (7.60-12.5)
1940-1949	3.05 (2.08-4.48)	3.53 (2.78-4.49)	0.37 (0.13-1.04)	2.55 (1.93-3.35)
1950-1959	1.84 (1.25-2.72)	2.09 (1.64-2.67)	1.68 (0.93-3.03)	1.30 (0.98-1.73)
FHR (80%CI)				
1910-1939	9.27 (6.46-13.3)	6.82 (5.35-8.69)	3.28 (1.69-6.36)	9.38 (7.27-12.1)
1940-1949	3.05 (2.08-4.48)	3.44 (2.70-4.39)	0.40 (0.14-1.11)	2.49 (1.88-3.28)
1950-1959	1.84 (1.25-2.72)	2.07 (1.63-2.65)	1.75 (0.96-3.17)	1.29 (0.97-1.72)
BIC	2,588.21	6,312.98	586.03	4,864.95
Macrorregion of residence				
Southeast (reference)	1.4 (1.0-1.9)	4.0 (3.3-4.7)	0.3 (0.1-0.5)	3.1 (2.3-3.8)
North	3.5 (1.2-5.8)	2.7 (1.5-3.9)	0.3 (0.0-0.5)	3.8 (2.1-5.5)
Northeast	2.6 (1.6-3.5)	5.1 (3.6-6.6)	0.5 (0.2-0.8)	4.7 (4.0-5.5)
South	1.7 (0.9-2.6)	4.2 (2.7-5.6)	0.4 (0.1-0.7)	3.3 (2.3-4.4)
Central-West	2.4 (1.2-3.7)	5.5 (3.4-7.6)	0.7 (0.1-1.3)	1.6 (0.7-2.4)
p-value ^##^	0.076	0.074	0.468	0.001
HR (80%CI)				
North	1.20 (0.79-1.80)	0.74 (0.54-0.99)	2.28 (1.06-4.93)	0.83 (0.61-1.14)
Northeast	1.39 (1.07-1.80)	1.08 (0.92-1.28)	1.51 (0.83-2.77)	1.28 (1.07-1.53)
South	1.14 (0.81-1.61)	1.02 (0.82-1.26)	2.00 (1.02-3.94)	1.00 (0.79-1.28)
Central-West	1.56 (1.11-2.20)	1.10 (0.87-1.39)	1.84 (0.85-3.970	0.52 (0.36-0.75)
FHR (80%CI)				
North	1.17 (0.77-1.78)	0.71 (0.52-0.96)	2.23 (1.03-4.86)	0.76 (0.55-1.05)
Northeast	1.33 (1.02-1.74)	1.00 (0.84-1.19)	1.52 (0.83-2.81)	1.11 (0.92-1.33)
South	1.12 (0.79-1.59)	1.01 (0.81-1.25)	1.96 (0.99-3.88)	1.01 (0.79-1.29)
Central-West	1.54 (1.09-2.18)	1.07 (0.84-1.35)	1.82 (0.84-3.94)	0.49 (0.34-0.71)
BIC	2,702.37	6,464.27	596.98	5,082.84
Residential zone				
Urban (reference)	2.1 (1.5-2.7)	4.4 (3.7-5.2)	0.4 (0.2-0.6)	3.4 (2.9-4.0)
Rural	2.7 (1.6-3.8)	4.0 (2.5-5.4)	0.6 (0.2-0.9)	3.4 (2.4-4.4)
p-value ^##^	0.307	0.593	0.333	0.952
HR (80%CI)	1.21 (0.92-1.59)	0.89 (0.73-1.08)	2.47 (1.52-4.03)	0.95 (0.77-1.18)
FHR (80%CI)	1.11 (0.84-1.46)	0.75 (0.62-0.92)	2.45 (1.49-4.03)	0.77 (0.62-0.96)
BIC	2,691.74	6,448.16	584.58	5,077.22
Marital status				
Married (reference)	1.5 (1.1-1.9)	3.5 (2.8-4.1)	0.3 (0.1-0.4)	2.2 (1.7-2.7)
Unmarried	3.0 (1.9-4.2)	5.3 (4.1-6.5)	0.6 (0.3-0.9)	4.8 (3.8-5.7)
p-value ^##^	0.002	0.006	0.053	< 0.001
HR (80%CI)	2.02 (1.63-2.50)	1.53 (1.33-1.75)	1.81 (1.15-2.86)	2.13 (1.82-2.49)
FHR (80%CI)	2.15 (1.72-2.67)	1.66 (1.44-1.92)	2.23 (1.40-3.56)	2.21 (1.87-2.59)
BIC	2,674.73	6,431.46	588.01	5,038.45
Livng alone				
No (reference)	2.2 (1.5-2.9)	4.4 (3.6-5.2)	0.4 (0.2-0.5)	3.3 (2.8-3.8)
Yes	2.0 (0.9-3.2)	4.2 (2.6-5.7)	0.8 (0.1-1.5)	4.2 (3.0-5.5)
p-value ^##^	0.780	0.813	0.072	0.098
HR (80%CI)	1.01 (0.73-1.40)	1.14 (0.93-1.39)	2.04 (1.18-3.53)	1.54 (1.26-1.89)
FHR (80%CI)	1.03 (0.74-1.42)	1.14 (0.93-1.40)	2.27 (1.30-3.94)	1.54 (1.25-1.89)
BIC	2,692.93	6,449.31	587.66	5,071.62
Has health insurance				
No (reference)	2.4 (1.7-3.1)	4.4 (3.6-5.2)	0.5 (0.3-0.7)	3.4 (2.9-3.9)
Yes	1.4 (0.8-2.1)	4.1 (3.1-5.0)	0.2 (0.0-0.5)	3.4 (2.5-4.4)
p-value ^##^	0.050	0.560	0.289	0.924
HR (80%CI)	0.71 (0.54-0.93)	0.99 (0.84-1.16)	0.33 (0.15-0.72)	1.01 (0.84-1.20)
FHR (80%CI)	0.76 (0.57-1.00)	1.13 (0.95-1.34)	0.35 (0.16-0.77)	1.21 (1.00-1.47)
BIC	2,687.80	6,431.68	583.89	5,042.00
Home covered by FHS				
Yes (reference)	2.4 (1.6-3.2)	4.4 (3.5-5.3)	0.4 (0.2-0.5)	3.7 (3.2-4.2)
No	1.8 (1.1-2.4)	4.2 (3.0-5.3)	0.5 (0.1-1.0)	3.0 (2.0-3.9)
p-value ^##^	0.214	0.784	0.575	0.180
HR (80%CI)	0.79 (0.61-1.02)	0.95 (0.81-1.12)	0.69 (0.38-1.24)	0.78 (0.65-0.94)
FHR (80%CI)	0.82 (0.63-1.07)	1.03 (0.87-1.21)	0.73 (0.41-1.31)	0.84 (0.70-1.02)
BIC	2,510.24	6,101.56	549.07	4,791.61
Number of morbidities ^§^				
None	1.3 (0.6-2.0)	3.1 (1.6-4.5)	0.5 (0.0-1.1)	1.4 (0.6-2.2)
One	1.8 (1.0-2.6)	3.9 (2.7-5.0)	0.4 (0.1-0.7)	2.7 (1.8-3.7)
Two or more (reference)	2.5 (1.7-3.2)	4.8 (4.0-5.6)	0.4 (0.2-0.6)	4.1 (3.5-4.8)
p-value ^##^	0.023	0.101	0.872	< 0.001
HR (80%CI)				
None	0.51 (0.35-0.74)	0.58 (0.46-0.73)	1.17 (0.61-2.25)	0.50 (0.38-0.66)
One	0.60 (0.44-0.80)	0.84 (0.71-0.99)	1.09 (0.61-1.94)	0.64 (0.52-0.79)
FHR (80%CI)				
None	0.49 (0.33-0.72)	0.55 (0.43-0.70)	1.06 (0.55-2.04)	0.49 (0.37-0.64)
One	0.58 (0.43-0.78)	0.80 (0.68-0.96)	1.02 (0.57-1.81)	0.64 (0.52-0.78)
BIC	2,497.82	6,168.10	537.03	4,867.21
Occurrence of fall in the last 12 months				
No (reference)	2.2 (1.6-2.8)	4.1 (3.3-4.8)	0.5 (0.3-0.7)	3.3 (2.7-3.8)
Yes	2.2 (1.1-3.4)	5.3 (4.0-6.6)	0.2 (0.0-0.4)	3.9 (2.9-4.9)
p-value ^##^	0.971	0.055	0.137	0.239
HR (80%CI)	1.00 (0.78-1.28)	1.40 (1.20-1.62)	0.62 (0.33-1.15)	1.45 (1.23-1.71)
FHR (80%CI)	1.00 (0.78-1.29)	1.42 (1.22-1.65)	0.64 (0.34-1.20)	1.45 (1.23-1.72)
BIC	2,692.68	6,441.08	587.12	5,070.00
Limitation in BADL ^§§^				
No (reference)	1.6 (1.2-2.0)	3.6 (3.0-4.3)	0.4 (0.2-0.6)	2.2 (1.8-2.5)
Yes	5.0 (2.8-7.2)	7.7 (5.9-9.4)	0.6 (0.2-1.1)	9.4 (7.4-11.4)
p-value ^##^	< 0.001	< 0.001	0.255	< 0.001
HR (80%CI)	2.95 (2.36-3.68)	2.74 (2.38-3.17)	1.49 (0.86-2.58)	4.07 (3.49-4.75)
FHR (80%CI)	2.87 (2.30-3.58)	2.60 (2.25-3.01)	1.47 (0.85-2.55)	3.78 (3.23-4.42)
BIC	2,650.99	6,382.46	587.50	4,965.06
Smoking habit				
Never smoked (reference)	2.1 (1.3-2.9)	3.6 (2.8-4.3)	0.2 (0.0-0.3)	2.7 (2.0-3.5)
Ex-smoker	2.2 (1.4-2.9)	4.9 (3.7-6.2)	0.5 (0.3-0.8)	4.4 (3.2-5.6)
Smoket	2.5 (1.5-3.4)	5.2 (3.7-6.6)	0.8 (0.2-1.5)	3.1 (2.0-4.2)
p-value ^##^	0.823	0.048	0.030	0.058
HR (80%CI)				
Ex-smoker	1.15 (0.91-1.46)	1.47 (1.26-1.71)	3.47 (1.89-6.38)	1.68 (1.42-2.00)
Smoker	1.37 (1.03-1.82)	1.57 (1.30-1.89)	4.14 (2.11-8.13)	1.46 (1.17-1.82)
FHR (80%CI)				
Ex-smoker	1.09 (0.86-1.38)	1.33 (1.14-1.56)	3.29 (1.79-6.08)	1.57 (1.32-1.87)
Smoker	1.30 (0.97-1.73)	1.45 (1.20-1.75)	3.91 (1.98-7.70)	1.36 (1.09-1.71)
BIC	2,695.25	6,444.87	581.02	5,070.48
Alcohol use				
No (reference)	2.4 (1.7-3.1)	4.8 (4.0-5.7)	0.4 (0.2-0.5)	3.8 (3.3-4.4)
Yes	1.6 (1.0-2.3)	2.9 (2.1-3.8)	0.7 (0.2-1.1)	2.1 (1.5-2.8)
p-value ^##^	0.052	0.002	0.172	< 0.001
HR (80%CI)	0.70 (0.54-0.91)	0.71 (0.60-0.84)	1.04 (0.63-1.73)	0.59 (0.49-0.72)
FHR (80%CI)	0.71 (0.55-0.93)	0.71 (0.60-0.85)	0.97 (0.58-1.62)	0.63 (0.51-0.77)
BIC	2,688.47	6,441.79	590.09	5,048.69
Physical activity practice ^§§§^				
Sufficient (reference)	1.5 (1.0-1.9)	3.1 (2.5-3.8)	0.5 (0.3-0.7)	1.8 (1.4-2.1)
Insufficient	3.9 (2.4-5.5)	7.0 (5.6-8.5)	0.4 (0.2-0.7)	6.7 (5.4-8.0)
p-value ^##^	< 0.001	< 0.001	0.723	< 0.001
HR (80%CI)	2.66 (2.15-3.30)	2.17 (1.88-2.50)	1.04 (0.64-1.71)	3.15 (2.68-3.71)
FHR (80%CI)	2.59 (2.08-3.21)	2.08 (1.80-2.40)	1.05 (0.64-1.72)	3.15 (2.68-3.71)
BIC	2,542.39	5,897.26	565.77	4,579.91

80%CI: 80% confidence interval; 95%CI: 95% confidence interval; BADL: basic ativities of daily living; BIC: Bayesian information criterion; FHS: Family Health Strategy.

* HR refers to the univariate (crude) analysis considering the complex sample design; FHR refers to the univariate model with random effect (frailty) for the 32 intersectional strata, constructed based on the combination of sex, race/skin color, education, and income;

** Group 1: infectious and parasitic diseases, malnutrition, and reproductive health problems;

*** Group 2: non-communicable chronic diseases (NCDs), neoplasms, and their risk factors;

^#^ Group 3: External causes and mental/behavioral disorders;

^##^ p-value for the weighted Kaplan-Meier test;

^###^ p-value for Mood’s median test;

^§^ Number of self-reported morbidities: systemic arterial hypertension; back problem; high cholesterol; arthritis or rheumatism; depression; diabetes; heart problem (angina, heart attack, heart failure); emphysema, chronic bronchitis, or chronic obstructive pulmonary disease (COPD); stroke; cancer; asthma; chronic kidney failure; Parkinson’s; and Alzheimer’s;

§§ Functional limitation: any difficulty in BADL - dressing, eating, bathing, using the toilet, moving around (walking within a room), and getting out of bed;

§§§ Physical activity practice: sufficient - performing at least 150 minutes of physical activity per week; insufficient - performing less than 150 minutes of physical activity per week.

In the final model adjusted by cause groups (Table 4), age was a consistent risk factor for groups 1 (infectious diseases) and GC. Not being married increased the risk of death from group 1 causes and, more sharply, from external causes (group 3) (FHR = 2.14). Functional limitation was associated with mortality from groups 1 and 2, but not from group 3. Smoking emerged as a transversal risk factor, significantly increasing all-cause mortality, noting a more than tripled risk of death from external causes (group 3) for both former smokers (FHR = 3.29) and current smokers (FHR = 3.65). Furthermore, residing in rural areas was associated with a 2.5 times higher risk of death from external causes, and the Central-West macro-region presented a significantly lower risk of registration of death from GC compared to the Southeast.

**Table 4:** Fragility hazard ratio (FHR) * and their respective 95% confidence intervals (95%CI) from Cox proportional hazards models adjusted by grouping of the 10th revision of the International Classification of Diseases (ICD-10) and classification as garbage code (GC). *Brazilian Longitudinal Study of Aging* (ELSI-Brasil, 2015-2021).

Variables	Group 1 **	Group 2 ***	Group 3 ^#^	GC
FHR (95%CI)	FHR (95%CI)	FHR (95%CI)	FHR (95%CI)
Age				
Continuous	1.06 (1.05-1.08)	-	-	1.07 (1.06-1.08)
p-value ^##^	< 0.001	-	-	< 0.001
Categorized age (years)				
Under 60	-	Reference	-	-
60 or more	-	2.82 (2.14-3.72)	-	-
p-value ^##^		< 0.001	-	-
Macrorregion of residence				
Southeast	-	-	-	Reference
North	-	-	-	0.71 (0.42-1.20)
p-value ^##^				0.200
Northeast	-	-	-	0.85 (0.64-1.15)
p-value ^##^				0.300
South	-	-	-	1.02 (0.69-1.50)
p-value ^##^				> 0.900
Central-West	-	-	-	0.39 (0.21-0.71)
p-value ^##^				0.002
Marital sataus				
Married	Reference	-	Reference	-
Unmarried	1.41 (1.00-2.00)	-	2.14 (1.05-4.39)	-
p-value ^##^	0.052	-	0.037	-
Limitation in BADL ^###^				
No	Reference	Reference	Reference	-
Yes	1.82 (1.27-2.63)	2.29 (1.81-2.90)	-	-
p-value ^##^	0.001	< 0.001	-	-
Smoking habit				
Never smoked (base)	Reference	Reference	Reference	-
Ex-smoker	1.09 (0.75-1.58)	1.37 (1.07-1.76)	3.29 (1.29-8.40)	-
p-value ^##^	0.600	0.011	0.013	-
Smoker	1.80 (1.15-2.82)	1.69 (1.24-2.29)	3.65 (1.29-10.40)	-
p-value ^##^	0.011	< 0.001	0.015	-

95%CI: 95% confidence interval; BADL: basic ativities of daily living.

* Multiple Cox proportional hazards model with frailty for the 32 intersectional strata, constructed based on the combination of sex, race/skin color, education, and income;

** Group 1: infectious and parasitic diseases, malnutrition, and reproductive health problems;

*** Group 2: chronic non-communicable diseases (CNCDs), neoplasms, and their risk factors;

^#^ Group 3: external causes and mental/behavioral disorders;

^##^ p-value referring to the Cox proportional hazards test;

### Functional limitation: any difficulty in BADL - dressing, eating, bathing, using the toilet, moving aroung (walking within a room), and getting out of bed.

## Discussion

This study showed that social position, constructed at the intersection of multiple axes of oppression, is a fundamental determinant of mortality in older adults in Brazil. The application of an analytical approach that integrates intersectionality theory with survival models enabled quantifying how mortality risks are unequally distributed, producing vulnerabilities that cannot be understood by the sum of their components in isolation. Therefore, this work aligns with a political act of restoring denied humanities ^20^, making visible, through data, the realities that the hegemonic narrative has historically silenced ^14^ ^,^ ^21^. The use of whiteness as a reference group enables highlighting how structural racism hierarchizes the risk of death, establishing a universal reference of protection and privilege ^14^ ^,^ ^22^.

In parallel, it is important to note that the potential confounding effect of the COVID-19 pandemic was considered. However, preliminary analyses did not indicate a significant impact on the results, as the vast majority of observed deaths (over 98%) occurred in the pre-pandemic period, ensuring the reliability of the findings in relation to mortality patterns established before the health crisis. Thus, the proportional distribution of deaths showed an incomplete epidemiological transition, with chronic NCDs and neoplasms accounting for the largest share (41.8%), but with a significant persistence of infectious-parasitic diseases (21.2%) and external causes (4.1%), consistently with other analyses of the national situation ^23^ ^,^ ^24^.

The MAIHDA model without adjustment variables showed that 7% of the total variation in the probability of death was attributable to differences between the intersectional strata. In Public Health studies, such between-group variation of this magnitude indicates that social position is not a secondary risk factor, but a central predictor of life/death outcomes ^25^. The persistence of high residual risks for women with low education, even after adjusting for morbidities, reinforces the hypothesis of the “matriarchy of misery” ^7^, in which the inscription of inequality on the body manifests through the confluence of oppressions of gender and class. On the other hand, the paradoxical finding of protection in non-white men with intermediate education suggests a critical threshold at which the accumulation of social capital enables a more effective navigation in an often hostile healthcare system ^26^.

However, the interpretation of these findings, especially those related to race/skin color, requires caution as to the survival bias. By establishing the initial follow-up point at 50 years of age, the research does not consider the early mortality that predominantly affects non-White individuals ^27^. From the perspective of necropolitics, structural racism operates as a mechanism of exclusion that subtracts a considerable portion of the non-White race/skin color population even before the data collection is initiated. This can, consequently, attenuate the magnitude of health inequities in the outcomes ^14^ ^,^ ^22^.

The estimates of the Cox models confirm a documented social gradient of mortality ^28^ ^,^ ^29^, but the analysis by specific causes provides a granular view of how inequities are inscribed in the body. Male overmortality, for example, was imperatively manifested in external causes. This finding reflects not only exposure to occupational risks and violence, but also a social construction of masculinity that neglects self-care and prevention ^30^. Similarly, the accentuated risk of death from infectious diseases in the oldest birth cohort (1910-1939) is the empirical translation of the Life Course Epidemiology, reflecting a history of policies that neglected sanitation and nutrition for the majority of the population in past decades ^31^ ^,^ ^32^.

Physical frailty, represented by functional limitation in BADLs, emerged as a potent predictor of mortality, almost doubling the risk of death in the adjusted model. This outcome is not purely biological, but a product of life trajectories marked by inequity, being more common in groups with lower education and income, and aggravated by failures of the healthcare system in providing access to rehabilitation therapies ^33^ ^,^ ^34^. The strong association of functional limitation with mortality classified as GCs also suggests that the clinical complexity of frail individuals challenges the healthcare system’s capacity to assign a basic cause of death, resulting in unspecific classification ^35^.

The analysis of lifestyle variables shows the association between individual behaviors and structural health determinants. Smoking has been shown to be a persistent risk factor, with current smokers having an almost doubled risk of all-cause mortality. The attenuation of this effect by integrating the frailty model, however, indicates that a portion of the risk attributed to smoking is, in reality, mediated by the social vulnerability inherent in groups in which tobacco consumption is more prevalent ^36^.

The comparison between the standard and frailty Cox models demonstrated the limit of sectoral policies in the face of the strength of structural inequalities. The protective effect of the FHS, for example, was nullified in the frailty model. This result does not invalidate the merits of the FHS, but suggests that, for bodies burdened by multiple oppressions, access to primary health care may not be sufficient for overcoming the accumulated vulnerability ^37^ ^,^ ^38^. Contextual analysis also showed multiple “geographies of abandonment”, such as the 2.5 times higher risk of death from external causes in rural areas, challenging the narrative that concentrates violence in urban centers and indicating the higher lethality of events in areas with precarious health and transportation infrastructure ^39^ ^,^ ^40^.

However, it is emphasized that the observed differences between the standard Cox model and the frailty model suggest that the intersectional structure may absorb part of the inequalities previously attributed to contextual factors (such as area and macroregion of residence) that only emerge when controlling for the shared heterogeneity of the strata. However, the overlap of confidence intervals in the direct comparison between the different risk estimates for variables such as FHS and the North macrorregion demands caution, indicating that, although the inclusion of frailty alters statistical significance, the absolute magnitude of this shift should be interpreted from the perspective of accumulated structural vulnerability.

One of the findings with vast political implications is the alarming proportion of 33% of deaths classified as GC, corroborating previous analyses ^10^. This magnitude reveals a facet of data inequity that sustains structures of necropolitics ^22^, obscuring the true distribution of causes of death and leading to ineffective policies ^11^ ^,^ ^41^. The fact that the Central-West macrorregion presents a significantly lower risk of GC registration compared to the Southeast, even after adjustment, shows that improving data quality is a feasible political action, which depends on prioritizing strategies such as strengthening Death Verification Services and surveillance ^42^ ^,^ ^43^.

The strengths of the study include the use of a population-based cohort with national representativeness and the pioneering application of an analytical approach that integrates intersectionality theory with survival models. However, limitations should be considered. The main limitation is survival bias: by initiating follow-up at age 50 years, the cohort excludes the early mortality that disproportionately affects the non-white population, which likely underestimates the impact of structural racism ^44^. Other limitations include the use of self-reported data and the broad scope of the causes of death categories, which may conceal internal heterogeneities.

## Final considerations

The intersectional matrix built on sex, race/skin color, education, and income is a fundamental determinant of mortality in older adults in Brazil. The intersection of axes of oppression generates risks of death that are not reducible to the sum of their parts. This study contributes to knowledge by demonstrating, through a robust quantitative approach, that mortality inequalities are structural and persist even after controlling for individual and behavioral risk factors. The results reinforce the need for intersectoral public policies that act on the social structures that produce and perpetuate health inequities, going beyond interventions focused solely on the individual. By naming and measuring the impact of intersectionality, this research contributes to the construction of a critical, feminist, and anti-racist epidemiology, which not only describes the world but provides the tools to transform it.

## Data Availability

The sources of information used in the study are indicated in the body of the article.
