# Supplementary material for: Intersectionality in the mortality of Brazilians aged 50 years or older: analysis of ELSI-Brasil
Source: Cad Saude Publica. 2026 Oct 12;42:e00244825. doi: 10.1590/0102-311XEN244825 (PMC13678830; doi:10.1590/0102-311XEN244825)
Supplement: Translation

# Translation: Interseccionalidade na mortalidade de brasileiros com 50 anos ou mais: análise do ELSI-Brasil

**DOI:** 10.1590/0102-311XPT244825

**Authors:** Luiza Oliveira-Santos, Maria Fernanda Lima-Costa, Taynãna César Simões

**Affiliations:** 1 Instituto René Rachou, Fundação Oswaldo Cruz, Fiocruz, Belo Horizonte, Brasil.; 2 Núcleo de Estudos em Saúde Pública e Envelhecimento, Fundação Oswaldo Cruz, Fiocruz/Universidade Federal de Minas Gerais, Belo Horizonte, Brasil.

**Keywords:** Envelhecimento, Mortalidade, Interseccionalidade

## Abstract

O objetivo foi analisar os fatores associados à mortalidade geral e por grupos de causas específicas, incluindo códigos *garbage* (CG), sob a perspectiva da interseccionalidade (operacionalizada pela combinação de sexo, raça/cor da pele, escolaridade e renda), em uma coorte de brasileiros com 50 anos ou mais. Estudo de coorte prospectivo com dados de 8.918 participantes do *Estudo Longitudinal da Saúde dos Idosos Brasileiros* (ELSI-Brasil), acompanhados entre 2015 e 2021. A interseccionalidade foi operacionalizada em 32 estratos. A heterogeneidade na mortalidade foi quantificada pela Análise Multinível de Heterogeneidade Individual e Acurácia Discriminatória (MAIHDA). A associação com o tempo até o óbito foi analisada por meio de modelos de riscos proporcionais de Cox tradicional e com fragilidade, ajustados por variáveis sociodemográficas, de saúde e comportamentais. Ocorreram 817 óbitos. O modelo MAIHDA sem variáveis de ajuste indicou que 7% da variação na mortalidade foram atribuíveis às diferenças entre os estratos interseccionais. No modelo final ajustado com fragilidade, as variáveis de ajuste explicaram parte do risco, mas a análise dos resíduos revelou que estratos compostos por mulheres com baixa escolaridade mantiveram um risco de mortalidade residual elevado. Em contraste, homens não brancos de alta renda apresentaram risco inferior ao esperado. A interseccionalidade é um determinante estrutural da mortalidade. A sobreposição de eixos de opressão gera riscos que não são redutíveis à soma de suas partes, demandando políticas que atuem sobre as estruturas sociais que produzem iniquidade.

## Introdução

O envelhecimento populacional é um fenômeno demográfico global que, no Brasil, ocorre de forma acelerada e em um cenário de profundas e persistentes desigualdades sociais, impondo desafios singulares à saúde pública ^1^. O conhecimento científico atual indica que o país vivencia uma transição epidemiológica complexa e não linear, caracterizada pela tripla carga de doenças: a predominância crescente das doenças crônicas não transmissíveis (DCNT); a persistência de uma agenda não superada de doenças infectocontagiosas e nutricionais; e a manutenção de elevadas taxas de mortalidade por causas externas ^2^ ^,^ ^3^.

Sabe-se que a mortalidade em adultos mais velhos não se distribui de forma homogênea, sendo influenciada por uma complexa teia de fatores socioeconômicos, demográficos e de saúde acumulados ao longo da vida ^4^. Contudo, uma lacuna persistente na literatura é a tendência de analisar esses fatores de forma isolada, o que é insuficiente para capturar a complexidade das iniquidades em saúde. A pergunta de investigação que motiva este estudo é: como a sobreposição de múltiplos eixos de opressão social (gênero, raça/cor da pele, classe social) molda o risco de mortalidade geral e por causas específicas em adultos mais velhos no Brasil?

Para responder a essa questão, este estudo se baseia no modelo teórico da interseccionalidade. Nomeada por Kimberlé Crenshaw ^5^ em 1989, esse referencial, discutido por intelectuais brasileiras como Lélia Gonzalez ^6^ e Sueli Carneiro ^7^, sustenta que as opressões não atuam de forma aditiva, mas se reforçam mutuamente, produzindo iniquidades que são maiores do que a soma de suas partes.

Estudos internacionais evidenciam que indivíduos de coortes mais recentes, como os *baby boomers*, apresentam carga de multimorbidade superior a gerações anteriores, com desigualdades acentuadas por raça e gênero ^8^. No Brasil, idosos negros, especialmente as mulheres, possuem maior probabilidade de apresentar pior capacidade intrínseca - declínio físico e mental - quando comparados a homens brancos ^9^, evidenciando como a interseccionalidade molda o envelhecimento. Outra lacuna que justifica esta investigação é a metodológica: a alta prevalência de causas de óbito mal definidas ou códigos *garbage* (CG) nos registros oficiais brasileiros compromete a fidedignidade das análises e o planejamento de políticas, subestimando a carga de doenças em grupos vulnerabilizados ^10^ ^,^ ^11^.

Diante do exposto, o objetivo deste estudo foi analisar os fatores associados à mortalidade geral e por grupos de causas específicas, incluindo CG, considerando o papel da interseccionalidade nas desigualdades de risco e nessas associações, com base em uma coorte nacionalmente representativa de brasileiros com 50 anos ou mais, entre 2015 e 2021.

## Materiais e métodos

### Delineamento e população de estudo

Trata-se de um estudo de coorte prospectivo que utilizou os dados do *Estudo Longitudinal da Saúde dos Idosos Brasileiros* (ELSI-Brasil), uma investigação abrangente sobre o processo de envelhecimento no país. O ELSI-Brasil emprega um delineamento amostral complexo, com estratificação de municípios, e seleção de setores censitários e domicílios para garantir a representatividade nacional. Detalhes completos sobre a metodologia do ELSI-Brasil foram publicados anteriormente ^12^.

Os participantes foram recrutados na linha de base do estudo, entre 2015 e 2016. O seguimento para o desfecho óbito foi realizado até a segunda onda de coleta de dados (2019-2021), com o período de observação encerrado em 31 de dezembro de 2021. Os dados de mortalidade, incluindo data e causa básica do óbito, foram obtidos por meio de relacionamento probabilístico (*linkage*) da base de dados do ELSI-Brasil com o Sistema de Informações sobre Mortalidade (SIM) do Ministério da Saúde. O procedimento de *linkage* utilizou variáveis de identificação pessoal (nome, data de nascimento e nome da mãe) e incluiu uma etapa de revisão manual para o tratamento de homônimos e a confirmação dos registros, seguindo os protocolos institucionais descritos anteriormente ^4^ ^,^ ^12^. Esse procedimento assegurou a fidedignidade dos dados e a confidencialidade dos participantes.

Os critérios de elegibilidade para esta análise foram: ter participado da linha de base do ELSI-Brasil e possuir informações completas para as variáveis utilizadas na definição do constructo de interseccionalidade. A amostra inicial do ELSI-Brasil foi de 9.412 participantes. Após a exclusão de 494 indivíduos por dados faltantes nas variáveis de exposição, a amostra analítica final foi composta por 8.918 participantes.

### Variáveis de desfecho

O desfecho principal foi o tempo até o óbito por todas as causas (mortalidade geral). O tempo de seguimento foi calculado em dias, da data da entrevista na linha de base até a data do óbito ou a data final do acompanhamento para os participantes censurados. Adicionalmente, os óbitos foram classificados em quatro grupos mutuamente excludentes para as análises de causas específicas, com base na 10ª revisão da Classificação Internacional de Doenças (CID-10). O primeiro grupo, “Doenças infecto-parasitárias, desnutrição e problemas reprodutivos”, incluiu os capítulos I, X, XI, XII e XIV. O segundo, “DCNT, neoplasias e seus fatores de risco”, abrangeu os capítulos II, III, IV, VI, IX e XIII. O terceiro grupo, “Causas externas e transtornos mentais/comportamentais”, foi composto pelos capítulos V e XX. Finalmente, o quarto, “Códigos *Garbage* (CG)”, incluindo o capítulo XVIII (Causas mal definidas) e outros códigos considerados pouco informativos para a saúde pública, conforme a lista do estudo *Global Burden of Disease* (GBD) ^13^.

### Estratégia analítica interseccional e variáveis de ajuste

A perspectiva interseccional foi adotada como estratégia analítica central, sendo operacionalizada pela combinação de quatro marcadores sociais para formar 32 estratos interseccionais: sexo biológico (feminino, masculino), raça/cor da pele autorreferida (branca, não branca), escolaridade (categorizada em quatro níveis: sem escolaridade formal, 1 a 4 anos, 5 a 10, e 11 anos ou mais) e renda domiciliar *per capita* (agrupada em até R$1.190,00 e maior que R$1.190,00).

Justifica-se a dicotomização da raça/cor da pele em “branca” e “não branca” (pardos, pretos, indígenas e amarelos) com base no conceito de racismo estrutural, que identifica a branquitude como referencial normativo de privilégio ^7^ ^,^ ^14^. Embora indígenas (n = 42) e amarelos (n = 5) tenham origens distintas, o reduzido tamanho amostral impediria estimativas isoladas confiáveis, optando-se por agrupá-los no polo de subalternização racial.

As variáveis de ajuste foram selecionadas baseando-se na literatura como potenciais confundidores. Essas incluíram características sociodemográficas e contextuais, como coorte de nascimento (agrupada em quatro períodos de 1910 a 1966), idade (contínua), macrorregião geográfica (Norte, Nordeste, Sul, Centro-oeste, Sudeste), zona de residência (urbana, rural), situação conjugal (não casado, casado) e morar sozinho (sim, não). Fatores relacionados ao acesso e uso de serviços de saúde, como a posse de plano de saúde (sim, não) e a cobertura domiciliar pela Estratégia Saúde da Família - ESF (sim, não), também foram considerados.

Finalmente, foram incluídas as variáveis de condição de saúde e comportamentais: o número de morbidades autorreferidas (construído com base na contagem de diagnósticos de hipertensão, problema de coluna, colesterol elevado, artrite, depressão, diabetes, problema cardíaco, doença pulmonar obstrutiva crônica - DPOC -, acidente vascular cerebral - AVC -, câncer, asma, insuficiência renal, doença de Parkinson e Alzheimer), a ocorrência de quedas no último ano (sim, não), a presença de limitação funcional em pelo menos uma das atividades básicas da vida diária (ABVD) - vestir-se, alimentar-se, tomar banho, usar o banheiro, locomover-se (caminhar dentro de um cômodo) e levantar-se da cama -, o uso de álcool (sim, não), o hábito tabagista (ex-fumante, fumante atual, nunca fumou) e o nível de prática de atividade física (suficiente - se realizava pelo menos 150 minutos de atividade física por semana -, insuficiente - se realizava menos de 150 minutos de atividade física por semana).

### Análise estatística

Inicialmente, foi realizada a análise descritiva de características da amostra e das proporções de óbito. As associações das variáveis com a mortalidade foram testadas através do teste qui-quadrado com correção de Rao-Scott ^15^ e do teste de medianas de Mood para a idade contínua ^16^, incorporando os pesos amostrais e efeito de desenho. Além disso, foram computados os critérios de informação Bayesianos (BIC, acrônimo em inglês), para fins de ranqueamento de significância e utilização na definição da ordem de entrada das variáveis nos modelos múltiplos ^17^. Para quantificar a heterogeneidade na mortalidade atribuível à posição social, foi ajustado um modelo nulo de Análise Multinível de Heterogeneidade Individual e Acurácia Discriminatória (MAIHDA, acrônimo em inglês), ou seja, sem variáveis de ajuste. Esse é um modelo de regressão logística multinível em que os indivíduos (nível 1) são aninhados em seus respectivos estratos interseccionais (nível 2). O coeficiente de correlação intraclasse (ICC, acrônimo em inglês) foi calculado baseando-se nesse modelo para estimar a proporção da variação total na mortalidade que é atribuível às diferenças entre os estratos ^18^.

Para analisar o tempo até o evento, foram utilizados os modelos de riscos proporcionais de Cox, cujas medidas de associação foram estimadas por meio de *hazard ratios* (HR) e seus respectivos intervalos de 95% de confiança (IC95%). A estratégia central da análise foi o uso de modelos de Cox com fragilidade, mensurados por meio de *fragility hazard ratio* (FHR). Nessa técnica estatística, a “fragilidade” refere-se a um efeito aleatório atribuído ao constructo de interseccionalidade, formado pelos estratos de posição social (combinação de sexo, raça/cor da pele, escolaridade e renda), a fim de capturar a heterogeneidade não observada que influencia o risco de mortalidade ^18^. Essa abordagem estende o modelo de Cox tradicional e permite uma estimativa mais precisa dos efeitos das covariáveis e do próprio efeito contextual da interseccionalidade ^19^.

A estratégia de modelagem seguiu três passos: (1) análise univariada de Cox para todas as variáveis de ajuste; (2) seleção de variáveis com p < 0,20 para a inclusão no modelo múltiplo; (3) ajuste do modelo de Cox com fragilidade múltiplo final, considerando um nível de significância de 5% (p < 0,05). A suposição de riscos proporcionais foi verificada graficamente e por meio da análise dos resíduos de Schoenfeld. A variável “prática de atividade física” violou essa suposição e, portanto, foi utilizada para estratificar o modelo final de mortalidade geral. A mesma estratégia analítica foi aplicada para cada um dos quatro grupos de causas de óbito. Devido ao reduzido número de eventos em categorias específicas, os modelos de fragilidade com 32 estratos apresentaram instabilidade de convergência, optando-se pelo modelo tradicional ajustado. As análises foram realizadas no software R (http://www.r-project.org), versão 4.5.2. O ELSI-Brasil foi aprovado pelo Comitê de Ética em Pesquisa da Fundação Oswaldo Cruz, Minas Gerais (CAAE 34649814.3.0000.5091).

## Resultados

A amostra final de 8.918 participantes foi acompanhada por um período mediano de 4,8 anos (intervalo interquartil: 4,1-5,4 anos). Durante o seguimento, ocorreram 817 óbitos. A distribuição das causas de morte revelou que as DCNT e neoplasias (grupo 2) foram responsáveis por 41,8% dos óbitos, seguidas por 33% de CG, doenças infecto-parasitárias (grupo 1) com 21,2%, e causas externas (grupo 3) com 4,1%. A aplicação do modelo MAIHDA sem variáveis de ajuste, que avalia a variabilidade da mortalidade apenas entre os estratos interseccionais, mostrou que 7% (ICC = 0,07) da variação total na chance de óbito foram atribuíveis às diferenças entre os 32 grupos. Esse resultado indica uma heterogeneidade moderada, mas significativa, sugerindo que a posição social é um preditor relevante da mortalidade.

A análise univariada (Tabela 1) evidenciou um gradiente social, em que fatores como sexo masculino, baixa escolaridade, idade avançada, não ser casado, ter limitação funcional, maior número de morbidades, tabagismo e atividade física insuficiente estiveram associados a um maior risco de morte. No entanto, a comparação entre o modelo de Cox padrão (HR) e o modelo de Cox com fragilidade (FHR), que considera a heterogeneidade interseccional, revelou nuances importantes. Variáveis contextuais, como morar na zona rural ou na macrorregião Norte, não mostraram associação significativa no modelo padrão, mas apresentaram associação estatisticamente significativa como fatores protetores no modelo de fragilidade (zona rural - FHR = 0,82; IC95%: 0,72-0,93), embora com sobreposição dos intervalos de confiança entre as estimativas (HR e FHR). Inversamente, a cobertura pela ESF, observada como fator protetor no modelo padrão, perdeu sua significância estatística após o ajuste pela fragilidade interseccional.

**Tabela 1:** Incidências e *hazard ratio* (HR) * dos modelos de riscos proporcionais Cox e de fragilidade (FHR) * estimadas de mortalidade geral, segundo variáveis de exposição e de ajuste. *Estudo Longitudinal da Saúde dos Idosos Brasileiros* (ELSI-Brasil, 2015-2021).

Variável	Incidência na amostra (IC95%)	Incidência de óbitos (IC95%)	Valor de p **	HR (IC80%)	FHR (IC80%)	Valor de p ***
Sexo biológico			0,012			-
Masculino	40,5 (38,3-43,0)	11,5 (10,2-12,9)		1,35 (1,23-1,47)	-	
Feminino (referência)	59,5 (57,3-62,0)	9,7 (8,3-11,0)			-	
Raça/Cor da pele			0,542			-
Parda	50,1 (46,7-54,0)	10,7 (9,2-12,2)		1,02 (0,93-1,12)	-	
Preta	9,9 (8,6-11,0)	9,3 (6,9-11,7)		0,95 (0,80-1,11)	-	
Indígena e amarela	4,8 (1,5-15,0)	11,7 (8,8-14,7)		1,05 (0,81-1,35)	-	
Branca (referência)	35,2 (30,6-40,0)	10,2 (8,8-11,6)		-	-	
Raça/Cor da pele dicotomizada			0,728			
Não branca	35,2 (30,6-40,0)	10,5 (9,3-11,8)		1,01 (0,92-1,11)	-	
Branca (referência)	64,8 (59,8-69,0)	10,2 (8,8-11,6)		-	-	
Escolaridade (anos)			< 0,001			-
11 ou mais	6,8 (5,8-8,0)	5,9 (3,5-8,3)		0,38 (0,30-0,47)	-	
5 a 10	34,8 (32,3-37,0)	6,3 (5,3-7,3)		0,38 (0,33-0,43)	-	
1 a 4	39,2 (37,4-41,0)	12,1 (10,7-13,6)		0,73 (0,66-0,82)	-	
Sem escolridade formal (referência)	19,3 (16,7-22,0)	15,9 (13,1-18,6)			-	
Renda domiciliar *per capita* (R$) (quartis)			0,018			
Maior que 1.190,00	24,9 (21,9-28,2)	8,7 (7,3-10,3)		0,80 (0,70-0,91)	-	
788,10 a 1.190,00	20,5 (18,3-23,0)	9,6 (7,8-11,3)		0,93 (0,81-1,07)	-	
425,10 a 788,00	29,5 (27,8-31,4)	12,0 (10,1-13,9)		1,10 (0,98-1,24)	-	
0,00 a 425,00 (referência)	25,0 (20,7-29,9)	11,0 (9,4-12,6)			-	
Renda *per capita* agrupada			0,018			
Maior que 1.190,00	24,9 (21,9-28,0)	8,7 (7,3-10,2)		0,78 (0,70-0,87)	-	
Até 1.190,00 (referência)	75,1 (71,8-78,0)	11,0 (9,7-12,2)			-	
Faixa etária (anos)			< 0,001			0,150
< 60 (referência)	20,3 (18,2-22,0)	5,8 (4,6-7,1)		-	-	
60 a 69	32,6 (31,1-34,0)	7,2 (6,1-8,4)		1,11 (0,96-1,29)	1,09 (0,94-1,27)	
70 a 79	27,7 (25,9-30,0)	10,1 (8,5-11,9)		1,64 (1,41-1,90)	1,54 (1,32-1,80)	
80 a 89	15,6 (14,0-17,0)	18,7 (15,8-21,4)		3,28 (2,82-3,81)	2,92 (2,50-3,42)	
90 ou mais	3,9 (3,2-5,0)	30,5 (24,0-36,9)		5,25 (4,35-6,35)	4,51 (3,71-5,49)	
Idade			< 0,001			0,131
Contínua	63 (56-72)	74 (64-80)		1,07 (1,07-1,08)	1,07 (1,07-1,08)	
Coorte de nascimento			< 0,001			0,482
1910-1939	15,9 (14,1-18,0)	27,9 (24,6-31,2)		8,11 (6,98-9,42)	7,68 (6,57-8,97)	
1940-1949	25,9 (24,3-28,0)	11,3 (9,6-12,9)		2,87 (2,45-3,37)	2,80 (2,38-3,29)	
1950-1959	32,0 (30,5-34,0)	6,6 (5,5-7,7)		1,75 (1,49-2,05)	1,74 (1,48-2,04)	
1960-1966 (referência)	26,1 (23,8-29,0)	3,6 (2,7-4,5)				
Macrorregião de residência			0,065			0,041
Norte	13,6 (4,2-36,0)	10,2 (5,0-15,5)		0,89 (0,74-1,07)	0,82 (0,62-0,99)	
Nordeste	28,0 (19,1-39,0)	12,9 (10,5-15,3)		1,21 (1,09-1,35)	1,07 (0,96-1,20)	
Sul	10,2 (5,5-18,0)	9,7 (7,9-11,4)		1,06 (0,92-1,22)	1,05 (0,91-1,21)	
Centro-oeste	14,3 (8,5-23,0)	10,2 (7,0-13,4)		0,99 (0,84-1,17)	0,94 (0,80-1,11)	
Sudeste (referência)	33,9 (25,4-44,0)	8,8 (7,8-9,8)				
Zona de residência			0,884			0,390
Rural	16,4 (11,0-24,0)	10,7 (8,9-12,4)		1,02 (0,90-1,15)	0,82 (0,72-0,93)	
Urbana (referência)	83,6 (76,1-89,0)	10,4 (9,2-11,5)		-	-	
Estado civil			< 0,001			0,028
Não casado	48,2 (45,9-51,0)	13,7 (11,7-15,6)		1,81 (1,65-1,98)	2,00 (1,82-2,20)	
Casado (referência)	51,8 (49,4-54,0)	7,4 (6,6-8,2)		-	-	
Mora(va) sozinho			0,431			0,580
Sim	14,7 (13,5-16,0)	11,3 (9,0-13,5)		1,28 (1,13-1,45)	1,30 (1,14-1,47)	
Não (referência)	85,3 (84,0-86,0)	10,3 (9,1-11,4)		-	-	
Posse de plano de saúde			0,056			0,940
Sim	21,8 (18,5-25,0)	9,2 (7,6-10,8)		0,91 (0,82-1,01)	1,14 (1,01-1,28)	
Não (referência)	78,2 (74,6-82,0)	10,7 (9,6-11,8)		-	-	
Domicílio coberto pela ESF			0,093			0,270
Não	26,6 (22,0-32,0)	9,4 (8,0-10,8)		0,85 (0,76-0,95)	0,95 (0,85-1,06)	
Sim (referência)	73,4 (68,3-78,0)	10,9 (9,5-12,2)		-	-	
Número de morbidades ^#^			0,012			0,320
Nenhuma	9,7 (9,0-11,0)	4,6 (2,4-6,8)		0,56 (0,48-0,65)	0,52 (0,45-0,61)	
Uma	16,4 (15,3-18,0)	6,9 (4,8-9,0)		0,73 (0,65-0,82)	0,70 (0,63-0,79)	
Duas ou mais (referência)	73,9 (72,4-75,0)	11,3 (9,7-12,8)				
Ocorrência de queda nos últimos 12 meses			0,150			0,460
Sim	24,5 (23,3-26,0)	11,6 (9,5-13,8)		1,30 (1,18-1,44)	1,32 (1,20-1,46)	
Não (referência)	75,5 (74,4-77,0)	10,0 (9,0-11,1)		-	-	
Limitação nas ABVD ^##^			< 0,001			0,004
Sim	17,6 (16,3-19,0)	22,7 (19,0-26,4)		3,13 (2,85-3,44)	2,88 (2,62-3,17)	
Não (referência)	82,4 (80,9-84,0)	7,8 (7,1-8,5)		-	-	
Hábito tabagista			< 0,001			0,160
Ex-fumante	39,1 (36,6-42,0)	12,0 (10,4-13,6)		1,51 (1,37-1,67)	1,35 (1,22-1,50)	
Fumante atual	16,4 (15,4-17,0)	11,6 (9,4-13,7)		1,54 (1,36-1,75)	1,39 (1,22-1,58)	
Nunca fumou (referência)	44,6 (42,0-47,0)	8,6 (7,3-9,9)				
Uso de álcool			< 0,001			0,061
Sim	23,9 (22,0-26,0)	7,3 (6,1-8,6)		0,68 (0,61-0,76)	0,71 (0,63-0,79)	
Não (referência)	76,1 (74,0-78,0)	11,4 (10,1-12,7)		-	-	
Prática de atividade física ^###^			< 0,001			< 0,001
Insuficiente	34,3 (32,0-37,0)	18,1 (15,1-21,1)		2,49 (2,27-2,73)	2,33 (2,12-2,56)	
Suficiente (referência)	65,7 (63,3-68,0)	6,8 (6,0-7,6)		-	-	

ABVD: atividades básicas da vida diária; ESF: Estratégia Saúde da Família; IC80%: intervalo de 80% de confiança; IC95%: intervalo de 95% de confiança.

* HR refere-se à análise univariada (bruta) considerando o desenho amostral complexo; FHR refere-se ao modelo univariado com efeito aleatório (fragilidade) para os 32 estratos interseccionais, construídos com base na combinação de sexo, raça/cor da pele, escolaridade e renda;

** Valor de p referente ao teste de Kaplan-Meier ponderado;

*** Valor de p do teste de proporcionalidade dos riscos (apresentado apenas para o modelo de fragilidade FHR);

^#^ Número de morbidades dentre os autorrelatos de: hipertensão arterial sistêmica; problema de coluna; colesterol elevado; artrite ou reumatismo; depressão; diabetes; problema cardíaco (angina, infarto, insuficiência cardíaca); enfisema, bronquite crônica ou doença pulmonar obstrutiva crônica (DPOC); acidente vascular cerebral (AVC); câncer; asma; insuficiência renal crônica; doença de Parkinson; e Alzheimer;

## Limitação funcional: qualquer dificuldade nas ABVD - vestir-se, alimentar-se, tomar banho, usar o banheiro, locomover-se (caminhar dentro de um cômodo) e levantar-se da cama;

### Prática de atividade física: Suficiente - realizar pelo menos 150 minutos de atividade física por semana; Insuficiente - realizar menor de 150 minutos de atividade física por semana.

A análise dos efeitos residuais para o modelo de fragilidade múltiplo permitiu quantificar a variabilidade residual no risco de mortalidade geral entre os estratos após o controle para os fatores de ajuste (Tabela 2). A variância do efeito aleatório (σ² = 0,08; p < 0,001) mostrou a persistência de grupos com risco de mortalidade acima e abaixo da média de mortalidade no período, independentemente das condições de saúde e dos comportamentos individuais. Os estratos que apresentaram um risco de mortalidade significativamente abaixo do esperado foram: homens não brancos, com renda alta, com 5 a 10 anos de escolaridade; e homens não brancos, com renda alta, sem escolaridade formal. Em contrapartida, os estratos com risco significativamente acima da média esperada incluíram mulheres, com 1 a 4 anos de escolaridade: não brancas, com renda baixa; brancas, com renda alta; e não brancas, com renda alta. Permaneceram como fatores de risco independentes: idade avançada (FHR = 1,06 por ano; IC95%: 1,05-1,07), ter limitação funcional nas ABVD (FHR = 1,99; IC95%: 1,69-2,34), não ser casado (FHR = 1,48; IC95%: 1,26-1,73) e ser ex-fumante (FHR = 1,36; IC95%: 1,15-1,61) ou fumante atual (FHR = 1,93; IC95%: 1,57-2,39). Não ter morbidades foi um fator protetor (FHR = 0,71; IC95%: 0,55-0,91).

**Tabela 2:** Estimativas de risco de mortalidade geral segundo as variáveis selecionadas: modelo de Cox com fragilidade (FHR) * ajustado para o constructo da interseccionalidade. *Estudo Longitudinal da Saúde dos Idosos Brasileiros* (ELSI-Brasil, 2015-2021).

Variável	FHR	IC95%	Valor de p **
Estado conjugal			
Casado	Referência	Referência	Referência
Não casado	1,48	1,26-1,73	< 0,001
Número de morbidades ***			
Nenhuma	0,71	0,55-0,91	0,007
Uma	0,91	0,75-1,10	0,300
Duas ou mais	Referência	Referência	Referência
Idade			
Contínua	1,06	1,05-1,07	< 0,001
Limitação nas ABVD ^#^			
Não	Referência	Referência	Referência
Sim	1,99	1,69-2,34	< 0,001
Hábito tabagista			
Nunca fumou	Referência	Referência	Referência
Ex-fumante	1,36	1,15-1,61	< 0,001
Fumante	1,93	1,57-2,39	< 0,001
Variância (σ^2^)	0,08	-	< 0,001

ABVD: atividades básicas de vida diária; IC95%: intervalo de 95% de confiança.

* Modelo de riscos proporcionais de Cox múltiplo com fragilidade para os 32 estratos interseccionais, construídos com base na combinação de sexo, raça/cor da pele, escolaridade e renda;

** Valor de p referente ao teste de riscos proporcionais de Cox;

*** Número de morbidades autorreferidas: hipertensão arterial sistêmica; problema de coluna; colesterol elevado; artrite ou reumatismo; depressão; diabetes; problema cardíaco (angina, infarto, insuficiência cardíaca); enfisema, bronquite crônica ou doença pulmonar obstrutiva crônica (DPOC); acidente vascular cerebral (AVC); câncer; asma; insuficiência renal crônica; doença de Parkinson; e Alzheimer;

# Limitação funcional: qualquer dificuldade nas ABVD - vestir-se, alimentar-se, tomar banho, usar o banheiro, locomover-se (caminhar dentro de um cômodo) e levantar-se da cama.

A análise univariada por grupos de causas de óbito revelou padrões distintos. Para o grupo 1 (doenças infecciosas), coortes de nascimento mais antigas (HR = 9,27 para 1910-1939), residir no Nordeste (HR =1,39) ou Centro-oeste (HR = 1,56), não ser casado (HR = 2,02) e ter limitação funcional (HR = 2,95) foram fortes preditores de risco (Tabela 3). Para o grupo 2 (DCNT), além de um gradiente social por escolaridade, a limitação funcional (HR = 2,74) e o tabagismo (HR = 1,57 para fumantes) foram proeminentes. No grupo 3 (causas externas), ser do sexo masculino, residir na zona rural (HR = 2,47) e, principalmente, ter hábito tabagista (HR = 4,14 para fumantes) se destacaram como os principais fatores de risco. Por fim, para os óbitos por CG, ter limitação funcional apresentou o maior risco entre todos os demais fatores analisados (HR = 4,07), junto com idade avançada e baixa escolaridade, indicando um perfil de vulnerabilidade clínica e social associado à imprecisão do diagnóstico da causa de morte.

**Tabela 3:** Incidência de mortalidade e *hazard ratio* (HR) * dos modelos de riscos proporcionais Cox e de fragilidade (FHR) * por agrupamento da 10ª revisão da Classificação Internacional de Doenças (CID-10) e classificação como código *garbage* (CG), segundo variáveis de exposição e ajuste. *Estudo Longitudinal da Saúde dos Idosos Brasileiros* (ELSI-Brasil, 2015-2021).

Variáveis	Grupo 1 **	Grupo 2 ***	Grupo 3 #	CG
% (IC95%)	% (IC95%)	% (IC95%)	% (IC95%)
Modelo nulo	2,2 (1,7-2,9)	4,4 (3,7-5,1)	0,4 (0,3-0,6)	3,4 (3,0-4,0)
Sexo biológico				
Masculino (referência)	2,4 (1,7-3,2)	4,8 (3,8-5,7)	0,7 (0,3-1,0)	3,7 (3,0-4,3)
Feminino	2,0 (1,3-2,8)	4,1 (3,2-4,9)	0,3 (0,1-0,4)	3,3 (2,7-3,9)
Valor de p ^##^	0,408	0,250	0,031	0,304
HR (IC80%)	0,78 (0,63-0,96)	0,70 (0,61-0,80)	0,39 (0,24-0,63)	0,84 (0,72-0,98)
Raça/Cor da pele				
Branca (referência)	2,2 (1,5-3,0)	4,2 (3,4-5,1)	0,6 (0,3-0,8)	3,2 (2,4-3,9)
Não branca	2,2 (1,5-2,8)	4,4 (3,5-5,3)	0,4 (0,2-0,6)	3,6 (3,0-4,1)
Valor de p ^##^	0,856	0,706	0,252	0,384
HR (IC80%)	0,96 (0,77-1,19)	0,99 (0,86-1,13)	0,75 (0,47-1,17)	1,11 (0,95-1,30)
Renda *per capita* (R$)				
Até 1.190,00 (referência)	2,3 (1,7-3,0)	4,5 (3,7-5,4)	0,5 (0,3-0,7)	3,7 (3,1-4,2)
Maior que 1.190,00	1,8 (1,0-2,6)	3,9 (2,8-5,0)	0,3 (0,0-0,6)	2,8 (2,0-3,5)
Valor de p ^##^	0,264	0,372	0,399	0,055
HR (IC80%)	0,74 (0,58-0,96)	0,89 (0,76-1,04)	0,38 (0,19-0,76)	0,73 (0,61-0,88)
Escolaridade (anos)				
Sem escolaridade formal (referência)	3,5 (2,0-4,9)	6,6 (4,5-8,7)	6,6 (4,5-8,7)	5,5 (4,2-6,8)
1 a 4	2,6 (1,5-3,6)	5,1 (4,1-6,2)	5,1 (4,1-6,2)	4,0 (3,2-4,8)
5 a 10	1,4 (0,8-1,9)	2,3 (2,1-3,4)	2,8 (2,1-3,4)	1,8 (1,2-2,3)
11 ou mais	0,9 (0,2-1,7)	1,7 (0,6-2,8)	1,7 (0,6-2,8)	3,2 (1,1-5,2)
Valor de p ^##^	0,007	< 0,001	0,724	< 0,001
HR (IC80%)				
1 a 4	0,68 (0,53-0,88)	0,80 (0,67-0,95)	1,29 (0,67-2,49)	0,66 (0,55-0,79)
5 a 10	0,37 (0,27-0,50)	0,43 (0,35-0,52)	0,81 (0,40-1,63)	0,30 (0,24-0,38)
11 ou mais	0,34 (0,20-0,58)	0,36 (0,26-0,51)	0,38 (0,09-1,56)	0,41 (0,29-0,58)
Idade				
Contínua	73 (64-80)	71 (63-78)	62 (57-80)	77 (67-84)
Valor de p ^###^	< 0,001	< 0,001	0,900	< 0,001
HR (IC80%)	1,08 (1,07-1,09)	1,06 (1,06-1,07)	1,03 (1,00-1,05)	1,09 (1,08-1,10)
FHR (IC80%)	1,08 (1,07-1,09)	1,06 (1,06-1,07)	1,03 (1,01-1,05)	1,09 (1,08-1,10)
BIC	2.572,64	6.281,7	588,20	4.826,81
Coorte de nascimento				
1910-1939	0,7 (0,3-1,0)	1,5 (0,8-2,1)	0,4 (0,0-0,7)	1,01 (0,7-1,5)
1940-1949	6,2 (4,5-7,8)	8,9 (6,4-11,5)	1,0 (0,4-1,6)	11,8 (9,5-14,1)
1950-1959	2,3 (1,3-3,2)	5,8 (4,6-7,1)	0,1 (0,0-0,3)	3,1 (2,3-3,9)
1960-1966 (referência)	1,4 (0,9-2,0)	3,2 (2,5-3,9)	0,5 (0,2-0,7)	1,5 (1,0-2,0)
Valor de p ^##^	< 0,001	< 0,001	0,017	< 0,001
HR (IC80%)				
1910-1939	9,27 (6,46-13,3)	7,15 (5,64-9,07)	3,05 (1,60-5,82)	9,76 (7,60-12,5)
1940-1949	3,05 (2,08-4,48)	3,53 (2,78-4,49)	0,37 (0,13-1,04)	2,55 (1,93-3,35)
1950-1959	1,84 (1,25-2,72)	2,09 (1,64-2,67)	1,68 (0,93-3,03)	1,30 (0,98-1,73)
FHR (IC80%)				
1910-1939	9,27 (6,46-13,3)	6,82 (5,35-8,69)	3,28 (1,69-6,36)	9,38 (7,27-12,1)
1940-1949	3,05 (2,08-4,48)	3,44 (2,70-4,39)	0,40 (0,14-1,11)	2,49 (1,88-3,28)
1950-1959	1,84 (1,25-2,72)	2,07 (1,63-2,65)	1,75 (0,96-3,17)	1,29 (0,97-1,72)
BIC	2.588,21	6.312,98	586,03	4.864,95
Macrorregião de residência				
Sudeste (referência)	1,4 (1,0-1,9)	4,0 (3,3-4,7)	0,3 (0,1-0,5)	3,1 (2,3-3,8)
Norte	3,5 (1,2-5,8)	2,7 (1,5-3,9)	0,3 (0,0-0,5)	3,8 (2,1-5,5)
Nordeste	2,6 (1,6-3,5)	5,1 (3,6-6,6)	0,5 (0,2-0,8)	4,7 (4,0-5,5)
Sul	1,7 (0,9-2,6)	4,2 (2,7-5,6)	0,4 (0,1-0,7)	3,3 (2,3-4,4)
Centro-oeste	2,4 (1,2-3,7)	5,5 (3,4-7,6)	0,7 (0,1-1,3)	1,6 (0,7-2,4)
Valor de p ^##^	0,076	0,074	0,468	0,001
HR (IC80%)				
Norte	1,20 (0,79-1,80)	0,74 (0,54-0,99)	2,28 (1,06-4,93)	0,83 (0,61-1,14)
Nordeste	1,39 (1,07-1,80)	1,08 (0,92-1,28)	1,51 (0,83-2,77)	1,28 (1,07-1,53)
Sul	1,14 (0,81-1,61)	1,02 (0,82-1,26)	2,00 (1,02-3,94)	1,00 (0,79-1,28)
Centro-oeste	1,56 (1,11-2,20)	1,10 (0,87-1,39)	1,84 (0,85-3,970	0,52 (0,36-0,75)
FHR (IC80%)				
Norte	1,17 (0,77-1,78)	0,71 (0,52-0,96)	2,23 (1,03-4,86)	0,76 (0,55-1,05)
Nordeste	1,33 (1,02-1,74)	1,00 (0,84-1,19)	1,52 (0,83-2,81)	1,11 (0,92-1,33)
Sul	1,12 (0,79-1,59)	1,01 (0,81-1,25)	1,96 (0,99-3,88)	1,01 (0,79-1,29)
Centro-oeste	1,54 (1,09-2,18)	1,07 (0,84-1,35)	1,82 (0,84-3,94)	0,49 (0,34-0,71)
BIC	2.702,37	6.464,27	596,98	5.082,84
Zona de residência				
Urbana (referência)	2,1 (1,5-2,7)	4,4 (3,7-5,2)	0,4 (0,2-0,6)	3,4 (2,9-4,0)
Rural	2,7 (1,6-3,8)	4,0 (2,5-5,4)	0,6 (0,2-0,9)	3,4 (2,4-4,4)
Valor de p ^##^	0,307	0,593	0,333	0,952
HR (IC80%)	1,21 (0,92-1,59)	0,89 (0,73-1,08)	2,47 (1,52-4,03)	0,95 (0,77-1,18)
FHR (IC80%)	1,11 (0,84-1,46)	0,75 (0,62-0,92)	2,45 (1,49-4,03)	0,77 (0,62-0,96)
BIC	2.691,74	6.448,16	584,58	5.077,22
Estado civil				
Casado (referência)	1,5 (1,1-1,9)	3,5 (2,8-4,1)	0,3 (0,1-0,4)	2,2 (1,7-2,7)
Não casado	3,0 (1,9-4,2)	5,3 (4,1-6,5)	0,6 (0,3-0,9)	4,8 (3,8-5,7)
Valor de p ^##^	0,002	0,006	0,053	< 0,001
HR (IC80%)	2,02 (1,63-2,50)	1,53 (1,33-1,75)	1,81 (1,15-2,86)	2,13 (1,82-2,49)
FHR (IC80%)	2,15 (1,72-2,67)	1,66 (1,44-1,92)	2,23 (1,40-3,56)	2,21 (1,87-2,59)
BIC	2.674,73	6.431,46	588,01	5.038,45
Mora(va) sozinho				
Não (referência)	2,2 (1,5-2,9)	4,4 (3,6-5,2)	0,4 (0,2-0,5)	3,3 (2,8-3,8)
Sim	2,0 (0,9-3,2)	4,2 (2,6-5,7)	0,8 (0,1-1,5)	4,2 (3,0-5,5)
Valor de p ^##^	0,780	0,813	0,072	0,098
HR (IC80%)	1,01 (0,73-1,40)	1,14 (0,93-1,39)	2,04 (1,18-3,53)	1,54 (1,26-1,89)
FHR (IC80%)	1,03 (0,74-1,42)	1,14 (0,93-1,40)	2,27 (1,30-3,94)	1,54 (1,25-1,89)
BIC	2.692,93	6.449,31	587,66	5.071,62
Posse de plano de saúde				
Não (referência)	2,4 (1,7-3,1)	4,4 (3,6-5,2)	0,5 (0,3-0,7)	3,4 (2,9-3,9)
Sim	1,4 (0,8-2,1)	4,1 (3,1-5,0)	0,2 (0,0-0,5)	3,4 (2,5-4,4)
Valor de p ^##^	0,050	0,560	0,289	0,924
HR (IC80%)	0,71 (0,54-0,93)	0,99 (0,84-1,16)	0,33 (0,15-0,72)	1,01 (0,84-1,20)
FHR (IC80%)	0,76 (0,57-1,00)	1,13 (0,95-1,34)	0,35 (0,16-0,77)	1,21 (1,00-1,47)
BIC	2.687,80	6.431,68	583,89	5.042,00
Domicílio coberto por ESF				
Sim (referência)	2,4 (1,6-3,2)	4,4 (3,5-5,3)	0,4 (0,2-0,5)	3,7 (3,2-4,2)
Não	1,8 (1,1-2,4)	4,2 (3,0-5,3)	0,5 (0,1-1,0)	3,0 (2,0-3,9)
Valor de p ^##^	0,214	0,784	0,575	0,180
HR (IC80%)	0,79 (0,61-1,02)	0,95 (0,81-1,12)	0,69 (0,38-1,24)	0,78 (0,65-0,94)
FHR (IC80%)	0,82 (0,63-1,07)	1,03 (0,87-1,21)	0,73 (0,41-1,31)	0,84 (0,70-1,02)
BIC	2.510,24	6.101,56	549,07	47.91,61
Número de morbidades ^§^				
Nenhuma	1,3 (0,6-2,0)	3,1 (1,6-4,5)	0,5 (0,0-1,1)	1,4 (0,6-2,2)
Uma	1,8 (1,0-2,6)	3,9 (2,7-5,0)	0,4 (0,1-0,7)	2,7 (1,8-3,7)
Duas ou mais (referência)	2,5 (1,7-3,2)	4,8 (4,0-5,6)	0,4 (0,2-0,6)	4,1 (3,5-4,8)
Valor de p ^##^	0,023	0,101	0,872	< 0,001
HR (IC80%)				
Nenhuma	0,51 (0,35-0,74)	0,58 (0,46-0,73)	1,17 (0,61-2,25)	0,50 (0,38-0,66)
Uma	0,60 (0,44-0,80)	0,84 (0,71-0,99)	1,09 (0,61-1,94)	0,64 (0,52-0,79)
FHR (IC80%)				
Nenhuma	0,49 (0,33-0,72)	0,55 (0,43-0,70)	1,06 (0,55-2,04)	0,49 (0,37-0,64)
Uma	0,58 (0,43-0,78)	0,80 (0,68-0,96)	1,02 (0,57-1,81)	0,64 (0,52-0,78)
BIC	2.497,82	6.168,10	537,03	4.867,21
Ocorrência de queda nos últimos 12 meses				
Não (referência)	2,2 (1,6-2,8)	4,1 (3,3-4,8)	0,5 (0,3-0,7)	3,3 (2,7-3,8)
Sim	2,2 (1,1-3,4)	5,3 (4,0-6,6)	0,2 (0,0-0,4)	3,9 (2,9-4,9)
Valor de p ^##^	0,971	0,055	0,137	0,239
HR (IC80%)	1,00 (0,78-1,28)	1,40 (1,20-1,62)	0,62 (0,33-1,15)	1,45 (1,23-1,71)
FHR (IC80%)	1,00 (0,78-1,29)	1,42 (1,22-1,65)	0,64 (0,34-1,20)	1,45 (1,23-1,72)
BIC	2.692,68	6.441,08	587,12	5.070,00
Limitação nas ABVD ^§§^				
Não (referência)	1,6 (1,2-2,0)	3,6 (3,0-4,3)	0,4 (0,2-0,6)	2,2 (1,8-2,5)
Sim	5,0 (2,8-7,2)	7,7 (5,9-9,4)	0,6 (0,2-1,1)	9,4 (7,4-11,4)
Valor de p ^##^	< 0,001	< 0,001	0,255	< 0,001
HR (IC80%)	2,95 (2,36-3,68)	2,74 (2,38-3,17)	1,49 (0,86-2,58)	4,07 (3,49-4,75)
FHR (IC80%)	2,87 (2,30-3,58)	2,60 (2,25-3,01)	1,47 (0,85-2,55)	3,78 (3,23-4,42)
BIC	2.650,99	6.382,46	587,50	4.965,06
Hábito tabagista				
Nunca fumou (referência)	2,1 (1,3-2,9)	3,6 (2,8-4,3)	0,2 (0,0-0,3)	2,7 (2,0-3,5)
Ex-fumante	2,2 (1,4-2,9)	4,9 (3,7-6,2)	0,5 (0,3-0,8)	4,4 (3,2-5,6)
Fumante	2,5 (1,5-3,4)	5,2 (3,7-6,6)	0,8 (0,2-1,5)	3,1 (2,0-4,2)
Valor de p ^##^	0,823	0,048	0,030	0,058
HR (IC80%)				
Ex-fumante	1,15 (0,91-1,46)	1,47 (1,26-1,71)	3,47 (1,89-6,38)	1,68 (1,42-2,00)
Fumante	1,37 (1,03-1,82)	1,57 (1,30-1,89)	4,14 (2,11-8,13)	1,46 (1,17-1,82)
FHR (IC80%)				
Ex-fumante	1,09 (0,86-1,38)	1,33 (1,14-1,56)	3,29 (1,79-6,08)	1,57 (1,32-1,87)
Fumante	1,30 (0,97-1,73)	1,45 (1,20-1,75)	3,91 (1,98-7,70)	1,36 (1,09-1,71)
BIC	2.695,25	6.444,87	581,02	5.070,48
Uso de álcool				
Não (referência)	2,4 (1,7-3,1)	4,8 (4,0-5,7)	0,4 (0,2-0,5)	3,8 (3,3-4,4)
Sim	1,6 (1,0-2,3)	2,9 (2,1-3,8)	0,7 (0,2-1,1)	2,1 (1,5-2,8)
Valor de p ^##^	0,052	0,002	0,172	< 0,001
HR (IC80%)	0,70 (0,54-0,91)	0,71 (0,60-0,84)	1,04 (0,63-1,73)	0,59 (0,49-0,72)
FHR (IC80%)	0,71 (0,55-0,93)	0,71 (0,60-0,85)	0,97 (0,58-1,62)	0,63 (0,51-0,77)
BIC	2.688,47	6.441,79	590,09	5.048,69
Prática de atividade física ^§§§^				
Suficiente (referência)	1,5 (1,0-1,9)	3,1 (2,5-3,8)	0,5 (0,3-0,7)	1,8 (1,4-2,1)
Insuficiente	3,9 (2,4-5,5)	7,0 (5,6-8,5)	0,4 (0,2-0,7)	6,7 (5,4-8,0)
Valor de p ^##^	< 0,001	< 0,001	0,723	< 0,001
HR (IC80%)	2,66 (2,15-3,30)	2,17 (1,88-2,50)	1,04 (0,64-1,71)	3,15 (2,68-3,71)
FHR (IC80%)	2,59 (2,08-3,21)	2,08 (1,80-2,40)	1,05 (0,64-1,72)	3,15 (2,68-3,71)
BIC	2.542,39	5.897,26	565,77	4.579,91

ABVD: atividades básicas de vida diária; BIC: critério de informação Bayesiano; ESF: Estratégia Saúde da Família; IC80%: intervalo de 80% de confiança.

* HR refere-se à análise univariada (bruta) considerando o desenho amostral complexo; FHR refere-se ao modelo univariado com efeito aleatório (fragilidade) para os 32 estratos interseccionais, construídos com base na combinação;

** Grupo 1: doenças infecto-parasitárias, desnutrição e problemas de saúde reprodutiva;

*** Grupo 2: doenças crônicas não transmissíveis (DCNT), neoplasias e seus fatores de risco;

^#^ Grupo 3: causas externas e transtornos mentais/comportamentais;

^##^ Valor de p referente ao teste de Kaplan-Meier ponderado;

^###^ Valor de p referente ao teste de medianas de Mood;

^§^ Número de morbidades autorreferidas: hipertensão arterial sistêmica; problema de coluna; colesterol elevado; artrite ou reumatismo; depressão; diabetes; problema cardíaco (angina, infarto, insuficiência cardíaca); enfisema, bronquite crônica ou doença pulmonar obstrutiva crônica (DPOC); acidente vascular cerebral (AVC); câncer; asma; insuficiência renal crônica; doença de Parkinson; e Alzheimer;

§§ Limitação funcional: qualquer dificuldade nas ABVD - vestir-se, alimentar-se, tomar banho, usar o banheiro, locomover-se (caminhar dentro de um cômodo) e levantar-se da cama;

§§§ Prática de atividade física: suficiente (realizar pelo menos 150 minutos de atividade física por semana); insuficiente (realizar menos de 150 minutos de atividade física por semana).

No modelo final, ajustado por grupos de causas (Tabela 4), a idade foi um fator de risco consistente para os grupos 1 (doenças infecciosas) e CG. Não ser casado aumentou o risco de morte por causas do grupo 1 e, de forma mais acentuada, por causas externas (grupo 3) (FHR = 2,14). A limitação funcional esteve associada à mortalidade pelos grupos 1 e 2, mas não pelo grupo 3. O tabagismo emergiu como um fator de risco transversal, elevando significativamente a mortalidade por todas as causas definidas, com destaque para o risco mais que triplicado de morte por causas externas (grupo 3), tanto para ex-fumantes (FHR = 3,29) quanto para fumantes atuais (FHR = 3,65). Ademais, residir na zona rural esteve associado a um risco 2,5 vezes maior de morte por causas externas, enquato residir na macrorregião Centro-oeste apresentou um risco significativamente menor de registro de óbito por CG em comparação com a Sudeste.

**Tabela 4:** *Fragility hazard ratio* (FHR) * e seus respectivos intervalos de 95% de confiança (IC95%) dos modelos de riscos proporcionais Cox ajustados por agrupamento da 10ª revisão da Classificação Internacional de Doenças (CID-10) e classificação como código *garbage* (CG). *Estudo Longitudinal da Saúde dos Idosos Brasileiros* (ELSI-Brasil, 2015-2021).

Variáveis	Grupo 1 **	Grupo 2 ***	Grupo 3 ^#^	CG
FHR (IC95%)	FHR (IC95%)	FHR (IC95%)	FHR (IC95%)
Idade				
Contínua	1,06 (1,05-1,08)	-	-	1,07 (1,06-1,08)
Valor de p ^##^	< 0,001	-	-	< 0,001
Idade categorizada (anos)				
Menor que 60	-	Referência	-	-
60 ou mais	-	2,82 (2,14-3,72)	-	-
Valor de p ^##^		< 0,001	-	-
Macrorregião de residência				
Sudeste	-	-	-	Referência
Norte	-	-	-	0,71 (0,42-1,20)
Valor de p ^##^				0,200
Nordeste	-	-	-	0,85 (0,64-1,15)
Valor de p ^##^				0,300
Sul	-	-	-	1,02 (0,69-1,50)
Valor de p ^##^				> 0,900
Centro-oeste	-	-	-	0,39 (0,21-0,71)
Valor de p ^##^				0,002
Estado civil				
Casado	Referência	-	Referência	-
Não casado	1,41 (1,00-2,00)	-	2,14 (1,05-4,39)	-
Valor de p ^##^	0,052	-	0,037	-
Limitação nas ABDV ^###^				
Não	Referência	Referência	Referência	-
Sim	1,82 (1,27-2,63)	2,29 (1,81-2,90)	-	-
Valor de p ^##^	0,001	< 0,001	-	-
Hábito tabagista				
Nunca fumou (base)	Referência	Referência	Referência	-
Ex-fumante	1,09 (0,75-1,58)	1,37 (1,07-1,76)	3,29 (1,29-8,40)	-
Valor de p ^##^	0,600	0,011	0,013	-
Fumante	1,80 (1,15-2,82)	1,69 (1,24-2,29)	3,65 (1,29-10,40)	-
Valor de p ^##^	0,011	< 0,001	0,015	-

ABVD: atividades básicas de vida diária; IC95%: intervalo de 95% de confiança.

* Modelo múltiplo de riscos proporcionais de Cox com fragilidade para os 32 estratos interseccionais, construídos com base na combinação de sexo, raça/cor da pele, escolaridade e renda;

** Grupo 1: doenças infecto-parasitárias, desnutrição e problemas de saúde reprodutiva;

*** Grupo 2: doenças crônicas não transmissíveis (DCNT), neoplasias e seus fatores de risco;

^#^ Grupo 3: causas externas e transtornos mentais/comportamentais;

## Valor de p referente ao teste de riscos proporcionais de Cox;

### Limitação funcional: qualquer dificuldade nas ABVD - vestir-se, alimentar-se, tomar banho, usar o banheiro, locomover-se (caminhar dentro de um cômodo) e levantar-se da cama.

## Discussão

Este estudo mostrou que a posição social, construída na intersecção de múltiplos eixos de opressão, é um determinante fundamental da mortalidade em adultos mais velhos no Brasil. A aplicação de uma abordagem analítica que integra a teoria da interseccionalidade a modelos de sobrevivência permitiu quantificar como os riscos de morte são distribuídos de forma desigual, produzindo vulnerabilidades que não podem ser compreendidas pela soma isolada de seus componentes. Este trabalho, portanto, alinha-se a um ato político de restituir humanidades negadas ^20^, tornando visíveis, por meio de dados, as realidades que a narrativa hegemônica historicamente silencia ^14^ ^,^ ^21^. A utilização da branquitude como grupo de referência permite evidenciar como o racismo estrutural hierarquiza o risco de morte, estabelecendo um referencial universal de proteção e privilégio ^14^ ^,^ ^22^.

Paralelamente, é importante ressaltar que o potencial efeito de confundimento da pandemia de COVID-19 foi considerado. Contudo, análises preliminares não indicaram um impacto significativo nos resultados, uma vez que a grande maioria dos óbitos observados (mais de 98%) ocorreu no período pré-pandêmico, assegurando a confiabilidade dos achados em relação aos padrões de mortalidade estabelecidos antes da crise sanitária. Sendo assim, a distribuição proporcional dos óbitos revelou uma transição epidemiológica incompleta, com as DCNT e as neoplasias respondendo pela maior parcela (41,8%), mas com uma persistência significativa de doenças infecto-parasitárias (21,2%) e causas externas (4,1%), alinhando-se a outras análises do cenário nacional ^23^ ^,^ ^24^.

O modelo MAIHDA sem variáveis de ajuste revelou que 7% da variação total na probabilidade de óbito foram atribuíveis às diferenças entre os estratos interseccionais. Em estudos da área de Saúde Coletiva, uma variação intregrupos dessa magnitude indica que a posição social não é um fator de risco secundário, mas um preditor central do desfecho vida/morte ^25^. A persistência de riscos residuais elevados para mulheres de baixa escolaridade, mesmo após o ajuste por morbidades, reforça a hipótese do “matriarcado da miséria” ^7^, no qual a inscrição da desigualdade no corpo se manifesta pela confluência de opressões de gênero e classe. Por outro lado, o achado paradoxal de proteção em homens não brancos com escolaridade intermediária, sugere um limiar crítico em que o acúmulo de capital social permite navegar de forma mais eficaz em um sistema de saúde frequentemente hostil ^26^.

Contudo, a interpretação desses achados, especialmente relativos à raça/cor da pele, exige cautela quanto ao viés de sobrevivência. Ao estabelecer o marco inicial do seguimento aos 50 anos, a pesquisa não considera a mortalidade precoce que acomete majoritariamente pessoas de raça/cor da pele não branca ^27^. Sob o a perspectiva da necropolítica, o racismo estrutural opera como um mecanismo de exclusão que subtrai parte considerável da população de raça/cor da pele não branca antes mesmo do início da coleta. O que pode, consequentemente, atenuar a magnitude das iniquidades em saúde nos resultados ^14^ ^,^ ^22^.

As estimativas dos modelos de Cox confirmam um gradiente social de mortalidade já documentado ^28^ ^,^ ^29^, mas a análise por causas específicas oferece uma visão granular de como as iniquidades se inscrevem no corpo. A sobremortalidade masculina, por exemplo, foi manifestada de forma imperativa nas causas externas. Esse achado reflete não apenas a exposição a riscos ocupacionais e violência, mas também uma construção social da masculinidade que negligencia o autocuidado e a prevenção ^30^. Da mesma forma, o risco acentuado de morte por doenças infecciosas na coorte de nascimento mais antiga (1910-1939) é a tradução empírica da Epidemiologia do Curso de Vida, refletindo um histórico de políticas que negligenciaram o saneamento e a nutrição para a maioria da população em décadas passadas ^31^ ^,^ ^32^.

A fragilidade física, corporificada pela limitação funcional nas ABVD, emergiu como um potente preditor de mortalidade, quase duplicando o risco de óbito no risco ajustado. Esse desfecho não é puramente biológico, mas um produto de trajetórias de vida marcadas pela iniquidade, sendo mais comum em grupos de menor escolaridade e renda, e agravada por falhas do sistema de saúde em prover acesso a terapias de reabilitação ^33^ ^,^ ^34^. A forte associação da limitação funcional com a ocorrência de óbitos classificados como CG sugere, ainda, que a complexidade clínica de indivíduos frágeis desafia a capacidade do sistema de saúde de atribuir uma causa básica de óbito, resultando em uma classificação inespecífica ^35^.

A análise das variáveis de estilo de vida evidencia o nexo entre comportamentos individuais e os determinantes estruturais de saúde. O tabagismo mostrou ser um fator de risco persistente, com fumantes atuais apresentando um risco quase duplicado de mortalidade geral. A atenuação desse efeito ao integrar o modelo de fragilidade, todavia, sinaliza que uma parcela do risco atribuído ao hábito tabagista é, na realidade, mediada pela vulnerabilidade social inerente aos grupos onde o consumo de tabaco é mais prevalente ^36^.

A comparação entre os modelos de Cox padrão e de fragilidade demonstrou o limite das políticas setoriais frente à força das iniquidades estruturais. O efeito protetor da ESF, por exemplo, foi anulado no modelo de fragilidade. Esse resultado não invalida os méritos da ESF, mas sugere que, para os corpos que carregam o peso de múltiplas opressões, o acesso à atenção primária pode não ser suficiente para superar a vulnerabilidade acumulada ^37^ ^,^ ^38^. A análise contextual também revelou múltiplas “geografias de abandono”, como o risco 2,5 vezes maior de morte por causas externas na zona rural, desafiando a narrativa que concentra a violência nos centros urbanos e apontando para a maior letalidade de eventos em áreas com infraestrutura precária de saúde e transporte ^39^ ^,^ ^40^.

Salienta-se, contudo, que as diferenças observadas entre o modelo de Cox padrão e o modelo com fragilidade sugerem que a estrutura interseccional pode absorver parte das desigualdades anteriormente atribuídas a fatores contextuais (como zona e macrorregião de residência) que só emergem ao controlar a heterogeneidade compartilhada dos estratos. No entanto, a sobreposição dos intervalos de confiança na comparação direta entre as diferentes estimativas de risco para as variáveis como a ESF e macrorregião Norte demanda cautela, indicando que, embora a inclusão da fragilidade altere a significância estatística, a magnitude absoluta desse deslocamento deve ser interpretada sob a ótica da vulnerabilidade estrutural acumulada.

Um dos achados com vastas implicações políticas é a elevada proporção de 33% de óbitos classificados como CG, corroborando análises prévias ^10^. Essa magnitude revela uma faceta da iniquidade de dados que sustenta estruturas da necropolítica ^22^, ofuscando a verdadeira distribuição das causas de morte e levando a políticas ineficazes ^11^ ^,^ ^41^. O fato da macrorregião Centro-oeste apresentar um risco significativamente menor de registro de CG em comparação com a Sudeste, mesmo após o ajuste, evidencia que a melhoria da qualidade dos dados é uma ação política factível, que depende da priorização de estratégias como o fortalecimento dos Serviços de Verificação de Óbito (SVO) e da vigilância ^42^ ^,^ ^43^.

Os pontos fortes do estudo incluem o uso de uma coorte de base populacional com representatividade nacional e a aplicação pioneira de uma abordagem analítica que integra a teoria da interseccionalidade a modelos de sobrevivência. Contudo, limitações devem ser consideradas. A principal é o viés de sobrevivência: ao iniciar o seguimento aos 50 anos, a coorte exclui a mortalidade precoce que afeta desproporcionalmente a população não branca, o que provavelmente subestima o impacto do racismo estrutural ^44^. Outras limitações incluem a utilização de dados autorreferidos e a ampla abrangência das categorias de causas de óbito, que podem ocultar heterogeneidades internas.

## Considerações finais

A interseccionalidade construída com base em sexo, raça/cor da pele, escolaridade e renda é um determinante fundamental da mortalidade em adultos mais velhos no Brasil. A sobreposição de eixos de opressão gera riscos de morte que não se reduzem à soma de suas partes. Este estudo contribui para o conhecimento ao demonstrar, por meio de uma abordagem quantitativa sólida, que as desigualdades na mortalidade são estruturais e persistem mesmo após o controle por fatores de risco individuais e contextuais. Os resultados reforçam a necessidade de políticas públicas intersetoriais que atuem sobre as estruturas sociais que produzem e perpetuam a iniquidade em saúde, indo além de intervenções focadas apenas no indivíduo. Ao nomear e medir o impacto da interseccionalidade, esta pesquisa contribui para a construção de uma epidemiologia crítica, feminista e antirracista, que não apenas descreve o mundo, mas fornece as ferramentas para transformá-lo.

## Data Availability

As fontes de informação utilizadas no estudo estão indicadas no corpo do artigo.
